# A randomized controlled trial enhancing viral hepatitis testing in primary care via digital crowdsourced intervention

**DOI:** 10.1038/s41746-022-00645-2

**Published:** 2022-07-19

**Authors:** William C. W. Wong, Gifty Marley, Jingjing Li, Weihui Yan, Po-lin Chan, Joseph D. Tucker, Weiming Tang, Yuxin Ni, Dan Dan Cheng, Lou Cong, Wai-Kay Seto

**Affiliations:** 1 Department of Family Medicine & Primary Care, The University of Hong Kong-Shenzhen Hospital, Shenzhen, China; 2 Department of Family Medicine & Primary Care, Li Ka Shing Faculty of Medicine, The University of Hong Kong, Pok Fu Lam, Hong Kong; 3 The University of North Carolina Project-China, Global Health Center Office, 2nd Floor of Lao Gan Building, Yuexiu District, Guangzhou, China; 4 Social Entrepreneurship to Spur Health (SESH), 16th Floor of Lao Gan Building, No. 7 Lujing Road, Yuexiu District, Guangzhou, China; 5 World Health Organization Western Pacific Regional Office, Manila, The Philippines; 6 Institute of Global Health and Infectious Diseases, University of North Carolina, Chapel Hill, CA USA; 7 Faculty of Infectious and Tropical Diseases, LSHTM, London, UK; 8 Guangdong Second Provincial General Hospital, Guangzhou, China; 9 Department of Medicine, The University of Hong Kong-Shenzhen Hospital, Shenzhen, China; 10 Department of Medicine and State Key Laboratory of Liver Research, The University of Hong Kong, Hong Kong, China

**Keywords:** Viral hepatitis, Patient education

## Abstract

Despite the availability of hepatitis B virus (HBV) and hepatitis C virus (HCV) testing in primary care, testing rates in China remain low. Social media is an inexpensive means of disseminating information and could facilitate hepatitis testing promotion. We evaluated the capacity of digitally crowdsourced materials to promote HBV/HCV testing uptake via a randomized controlled trial (identifier: ChiCTR1900025771), which enrolled 750 Chinese primary care patients. We randomized patients (1:1) to receive crowdsourced HBV/HCV promotion materials through social media or facility-based care without promotional materials for four weeks. Exposure to all intervention materials was associated with increased odds of HBV (aOR = 1.79, 95% CI: 1.09–3.00) and HCV (aOR = 1.95, 95% CI: 1.29–2.99) testing compared to facility-based care. There was a significant reduction in hepatitis stigma among intervention group participants (HBV slope: −0.15, *p* < 0.05; and HCV slope: −0.13, *p* < 0.05). Digitally crowdsourced promotion messages could enhance hepatitis testing uptake and should be considered in hepatitis reduction strategies.

Trial registration: Chinese Clinical Trial Registry (ChiCTR1900025771) on September 9, 2019. Available from: http://www.chictr.org.cn/showproj.aspx?proj=42788

## Introduction

Hepatitis B virus (HBV) and hepatitis C virus (HCV) are associated with substantial morbidity and caused more than 1.4 million deaths in 2019^1^. China’s HBV and HCV disease burden accounts for one-third and 7% of global infections, respectively, with about 87 million people living with chronic HBV and about 9 million people living with HCV^2,3^. Testing as prevention remains an effective tool that facilitates early diagnosis and control of its spread, which translates to reduced transmissions and risks of chronic hepatitis infections^4,5^. However, only 19% of persons living with HBV and 18% of people living with HCV in China know their serostatus^6,7^. In the most recent publication, universal HBV screening in 18–70-year-old adults were found to be cost-effective and, initiated in 2021, this would save 3.46 million lives from HBV-related mortality in China^8^. Hence, innovative strategies are required to expand hepatitis testing in China to meet World Health Organization (WHO)’s goals of viral hepatitis elimination by 2030.

Studies have attributed the low test uptake rates to a lack of awareness and ineffective service promotion strategies^9,10^. However, awareness creation strategies still linger on paper-based promotion strategies in most lower/middle-income countries (LMICs), which is costly and has finite reach. The narrow concentration of hepatitis services delivery in health facilities, clinical protocol requirement that specialists (like gastroenterologists) undertake hepatitis treatment and the use of analog-based patient management systems have also contributed to substantial treatment delays, failed linkage-to-care, and patient loss-to-follow-up^10–12^. Digital health interventions, as the field of knowledge and practice associated with the development and use of digital technologies (like the internet of things, artificial intelligence, big data and robotics) to improve health, provides an exciting opportunity to enhance HBV and HCV test uptake^13^.

Digital tools like Electronic Health Record Systems (EHRs) can transform the paper-based management systems of health facilities to digitized systems to foster timely and higher quality patient care; hospitals could harness them to enhance successful linkage-to-care among patients^14,15^. Also, social media platforms are an inexpensive means of disseminating information to a broader audience within a short period and could enhance community engagement, awareness creation, and hepatitis testing promotion^16–18^. Furthermore, using community engagement strategies (like crowdsourcing) as a WHO recommended approach could help tailor test promotion materials for optimized impact^19^. But few studies have researched the potential role of digital tools in promoting hepatitis services uptake, especially in LMIC’s primary care settings. China is best suited to test this hypothesis due to the high rates of mobile technology use in the last decade. In China, WeChat is one of the most popular social media with 900 million daily users and 73.2% of them using it for social networking used it regularly everyday^20^.

Crowdsourcing is a community engagement approach to problem-solving which adopts the process of shifting problem-solving tasks from individuals and experts only to include the wider public, then implements selected solutions^21,22^. The WHO recognizes it as a valuable tool for soliciting new ideas in health research and the National Institutes of Health (NIH), and the National Academies of Sciences, Engineering, and Medicine (NASEM) have used it^21,23,24^. But only TDR Global (the Special Programme for Research and Training in Tropical Diseases) has pushed crowdsourcing forward globally and developed a guideline to standardize its use. In recent years, crowdsourcing has been extensively used worldwide and in China to optimize sexually transmitted infections (STIs) testing promotion and could be one of the most powerful tools needed to enhance hepatitis testing in China^25–27^. The current study evaluated the effects of crowdsourced intervention materials disseminated through social media on HBV and HCV test uptake among primary care patients in a tertiary hospital in urban China.

## Results

**Fig. 1:**
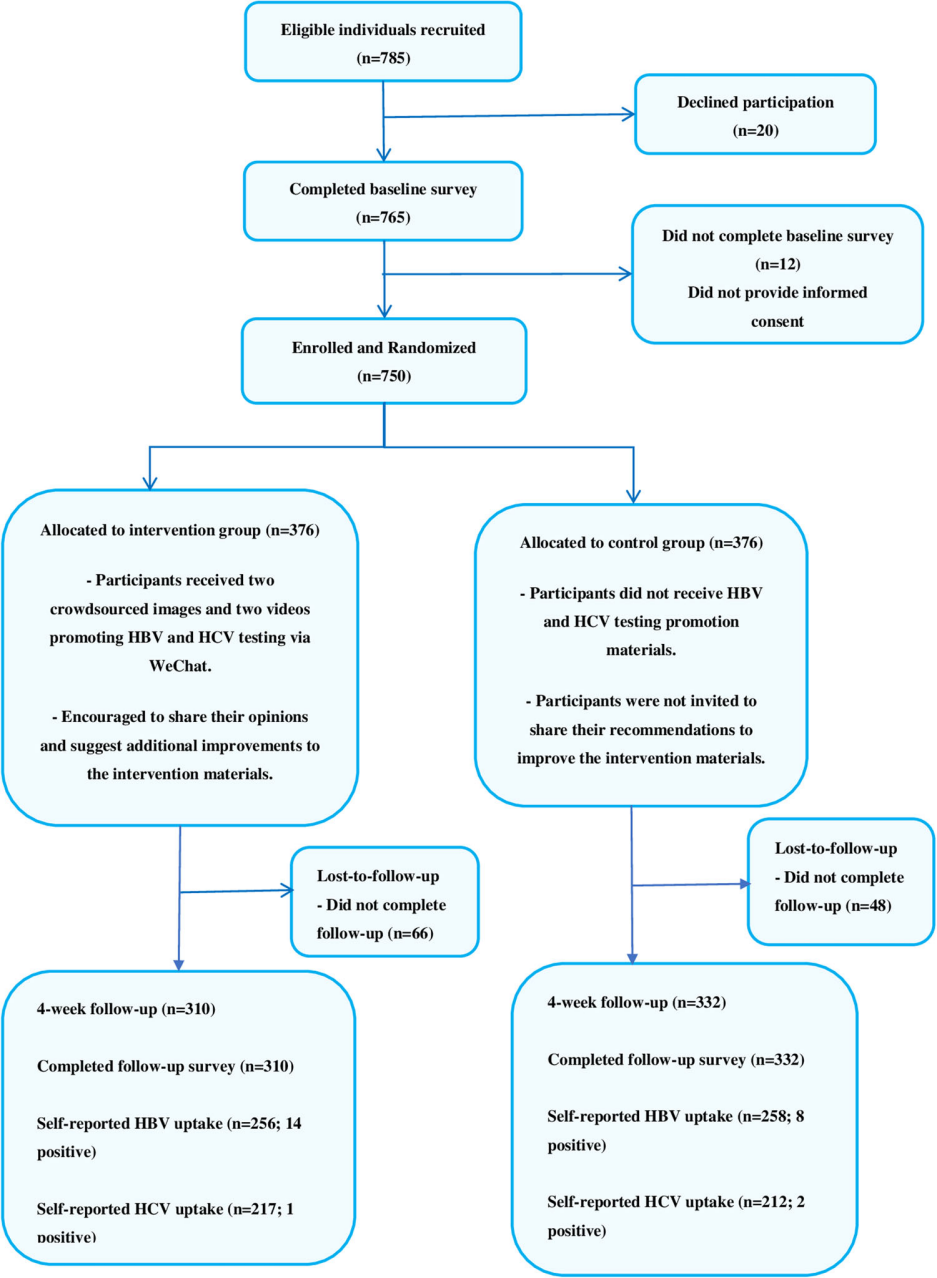
Study recruitment process. Flow chart showing participant recruitment, number of participants randomized to the intervention and control groups respectively, and number of participants retained in each group at follow-up.

Overall, we recruited 785 eligible patients from November 2019 until June 2021 of which 35 declined participation and 750 were enrolled. Using a generated random allocation sequence, 376 participants were randomly assigned to the intervention group and 374 to the control group. A total of 642/750 (85.6%) participants completed the follow-up survey at four weeks, with 17.6% (66/376) and 11.2% (42/374) lost-to-follow-up in the intervention and control groups, respectively. Study flow is detailed in Fig. 1.

### Baseline characteristics

The average participants’ age was 41years (±10.79) in the intervention group and 44 years (±11.46) in the control group. Most participants were married (intervention: 76.9%; *n* = 289; control: 79.7%, *n* = 298), heterosexuals (intervention: 98.4%, *n* = 370; control: 97.6%, *n* = 365), and reported an annual income above US$780 (intervention: 77.1%, *n* = 290; control: 69.3%, *n* = 259). More participants in the intervention group (39.9%, *n* = 150) than in the control group (32.4%, *n* = 121) had a bachelor’s degree, and more participants were institutionally employed in the intervention group (44.1%, *n* = 166) than in the control (39.0%, *n* = 146). About two-thirds of all participants (77.5%, *n* = 581) visited the HKU-SZH for medical care and paid with National medical insurance (66.5%, *n* = 499) [Table 1].

**Table 1:** Descriptive summary showing the socio-demographic characteristics of 750 primary care users enrolled in an online randomized controlled trial to evaluate a crowdsourced intervention on improving hepatitis testing in China, 2019–2021.

	Intervention (*N* = 376)		Control (*N* = 374)	
	*n*	%	*n*	%
Gender				
Male	142	37.8	175	46.8
Female	233	62.0	198	52.9
Other (e.g., transgender)	1	0.3	1	0.3
Age (years)	41.42	10.79	44.01	11.46
Marital status				
Single	66	17.6	54	14.4
Married/ cohabited	289	76.9	298	79.7
Divorced/ separated	17	4.5	18	4.8
Widowed	4	1.1	4	1.1
Sexual orientation				
Heterosexual	370	98.4	365	97.6
Homosexual/ bisexual	2	0.5	4	1.1
Not sure/ other	4	1.1	5	1.3
Highest education attained				
Junior high school or below	54	14.4	76	20.3
Senior high school/ diploma	137	36.4	148	39.6
Bachelor degree	150	39.9	121	32.4
Masters’ degree/Ph.D. or above	35	9.3	29	7.8
Employment status				
Student/ housewife	60	16.0	44	11.8
Self-employed	99	26.3	98	26.2
Employed	166	44.1	146	39.0
Public servant	5	1.3	10	2.7
Unemployed	10	2.7	10	2.7
Retired	36	9.6	66	17.6
Monthly income (USD)				
<780	86	22.9	115	30.7
780–1570	101	26.9	106	28.3
1571–2360	60	16.0	53	14.2
2361–3150	50	13.3	40	10.7
>3150	79	21.0	60	16.0
Most frequently visited places for medical care				
The University of Hong Kong-Shenzhen Hospital	285	75.8	296	79.1
Other hospitals in Shenzhen	63	16.8	49	13.1
Community health centers in Shenzhen	19	5.1	15	4.0
Medical institution outside of Shenzhen	9	2.4	14	3.7
Payment method				
National medical insurance	263	69.9	236	63.1
Corporate medical insurance	10	2.7	14	3.7
Other commercial medical insurance	11	2.9	13	3.5
Self-paid	92	24.5	111	29.7
Regular primary care practitioner	31	8.2	26	7.0

### Intervention exposure

Among the intervention group participants, 11.7% (44/376) saw up to 3 intervention materials and 61.4% (231/376) saw all four materials, and 26.9% (101/376) did not see any of the materials during the study period. Overall, 53.7% (202/376) of all participants provided feedback on the materials, and 17 ideas were sufficiently strong to be implemented.

### HBV and HCV test uptake

Overall, 68.5% (514/750) and 57.2 (429/750) of participants received HBV and HCV testing each, and 6.0% (45/750) self-reported HBV vaccination during the study period. Among the 236 HBV non-testers, not having enough time to test (31.2%, 75/236); not feeling at risk of HBV infection (29.7%, 70/236); and other unspecified reasons (33.1%, 78/236) were the most common reasons for not testing. Similarly, not having enough time to get tested 27.1% (87/321); did not feel at risk of HCV infection (32.4%;104/321); and did not know where to get testing (13.1%, 42/321) were the most common reasons for not receiving HCV testing among the 321 HCV non-testers during the study period (Supplementary Table 2).

### Intention-to-treat analysis

Overall, 68.1% (256/376) and 57.7% (217/376) of intervention group participants received HBV and HCV testing each by the end of the four weeks. Comparatively, 69.0% (258/374) and 56.7% (212/374) of the control group participants received HBV and HCV testing, respectively. The estimated odds of confirmed HBV and HCV test uptake were similar between the intervention and control arms using a missing = failure approach (OR = 1.20, 95% CI: 0.85,1.71) [Table 2].

**Table 2:** Intention-to-treat analysis of primary and secondary outcomes for an online randomized controlled trial to evaluate the impact of a crowdsourced intervention on hepatitis test uptake among 750 primary care users in urban China, 2019–2021.

Variables	Total (*N* = 750)	Intervention (*N* = 376)	Control (*N* = 374)	OR (95% CI)
	*n* (%)	*n* (%)	*n* (%)	
Primary outcome				
Confirmed HBV testing	514 (68.5)	256 (58.1)	258 (69.0)	1.23 (0.81–1.89)
Confirmed HCV testing	429 (57.2)	217 (57.7)	212 (56.7)	1.24 (0.87–1.76)
Confirmed both HBV and HCV test	375 (50.0)	186 (49.5)	189 (50.5)	1.20 (0.85–1.71)
Secondary outcome				
Confirmed HBV positives	22 (2.9)	14 (3.7)	8 (2.1)	1.77 (0.75–4.48)
Confirmed HCV positive	3 (0.4)	1 (0.3)	2 (0.5)	0.50 (0.02–5.20)
Sought medical care after receiving HBV results (self-report)	234 (32.1)	108 (29.0)	126 (33.4)	0.87 (0.62–1.22)
Sought medical care after receiving HCV results (self-report)	186 (25.5)	84 (22.5)	102 (27.1)	0.87 (0.61–1.24)
Self-reported HBV vaccination	40 (5.5)	22 (5.9)	18 (4.8)	1.32 (0.69–2.54)

All regressions used the control group as a reference. Multinomial regression was used to analyze confirmed HBV positive and confirmed HBV positive. Other outcomes were analyzed using binomial logistic regression. *p < 0.05, **p < 0.01. The 186 missing data were treated as missing = failure.

### Per-protocol analysis

A total of 14.4% (108/750) participants did not complete the follow-up surveys, and 11.1% (34/310) of completed follow-up surveys had data inconsistency. 35 of the 310 participants in the intervention group (11.3%) saw none of the intervention materials, 14.2% (44/310) saw at most three of the materials, and 74.2% (230/310) saw all four intervention materials within the 4 weeks’ study period. The odds of confirmed HBV and HCV test uptake were higher among fully exposed (aOR = 1.79, 95%. CI:1.09–3.00; and aOR = 1.95, 95% CI: 1.29–2.99, respectively) compared to non-exposed participants when using a complete case- approach. Similar increased odds of confirmed HBV and HCV testing among fully exposed participants (aOR = 1.75, 95% CI: 1.07–2.93; and aOR = 1.89, 95% CI: 1.25–2.88, respectively) compared to non-exposed participants was observed using multiple imputations. Table 3 below shows the results of per-protocol analyses. Also, of the seven baseline variables that were prespecified as potential confounders and adjusted for in multivariable logistic regression, being married or living with a partner (aOR = 0.26, 95% CI: 0.07–0.76) and being widowed (aOR = 0.02, 95% CI: 0.00–0.94) significantly decreased the odds of HBV testing compared to being single in a sub-analysis (Supplementary Table 3).

**Table 3:** Per-protocol analysis evaluating the impact of a crowdsourced intervention on hepatitis testing uptake among 560 primary care users in urban China, 2019–2021.

	HBV testing				HCV testing			
	OR (95% CI)		aOR (95% CI)		OR (95% CI)		aOR (95% CI)	
Complete-case analysis (556)								
Control group	ref		ref		ref		ref	
Intervention group	1.22 (0.79–1.87)		1.24 (0.79–1.93)		1.23 (0.86–1.77)		1.23 (0.85–1.79)	
Intervention exposure								
Saw none of the materials	ref		ref		ref		ref	
Saw some materials ^a^	0.64 (0.31–1.41)		0.64 (0.30–1.45)		0.78 (0.40–1.57)		0.75 (0.37–1.54)	
Saw all the materials	1.68 (1.04–2.77 *)		1.79 (1.09–3.00 *)		1.90 (1.28–2.85)**		1.95 (1.29–2.99)**	
Multiple Imputation analysis (*N* = 560)								
Study groups								
Control group	ref		ref		ref		ref	
Intervention group	1.21 (0.79–1.86)		1.24 (0.80–1.93)		1.21 (0.85–1.73)		1.22 (0.84–1.76)	
Intervention exposure								
Saw none of the materials	ref		ref		ref		ref	
Saw some materials ^a^	0.65 (0.31–1.43)		0.64 (0.30–1.45)		0.78 (0.40–1.57)		0.74 (0.36–1.52)	
Saw all materials	1.65 (1.03–2.70)*		1.75 (1.07–2.93)*		1.82 (1.23–2.73)**		1.89 (1.25–2.88)**	

Adjusted variables include age, sex, sexual orientation, marital status, education, occupation, and monthly income.

*p < 0.05; **p < 0.001.

^a^Had seen some of the materials meaning that participants looked at promotional materials from one to three weeks.

### Stigma reduction

Overall, there was a significant decrease in hepatitis stigma among participants in the intervention (HBV mean stigma score = 2.36, 95% CI: −0.26, −0.03; HCV mean stigma score = 2.38, 95% CI: −0.25, 0.00) compared to the controlled group (Table 4). However, a per-protocol sub-analysis showed no significant difference in stigma reduction among participants who saw all the intervention materials compared to those who saw at some and those who saw none (Supplementary Table 4).

**Table 4:** Intention-to-treat analysis to evaluate the impact of a crowdsourced intervention on hepatitis stigma among 564 primary care users in urban China, 2019–2021.

	Total (*N* = 564)	Control (*N* = 290)	Intervention (*N* = 274)	Adjusted beta	95%CI
	Mean (SD)	Mean (SD)	Mean (SD)		
Average HBV Stigma score	2.44 (0.68)	2.51 (0.64)	2.36 (0.72)	−0.15	−0.26, −0.03*
Average HCV Stigma score	2.44 (0.75)	2.50 (0.71)	2.38 (0.78)	−0.13	−0.25, 0.00*

Linear regression was used to analyze average stigma score for HBV and average stigma score for HCV. *p < 0.05, **p < 0.01.

### Linkage-to-care

**Fig. 2:**
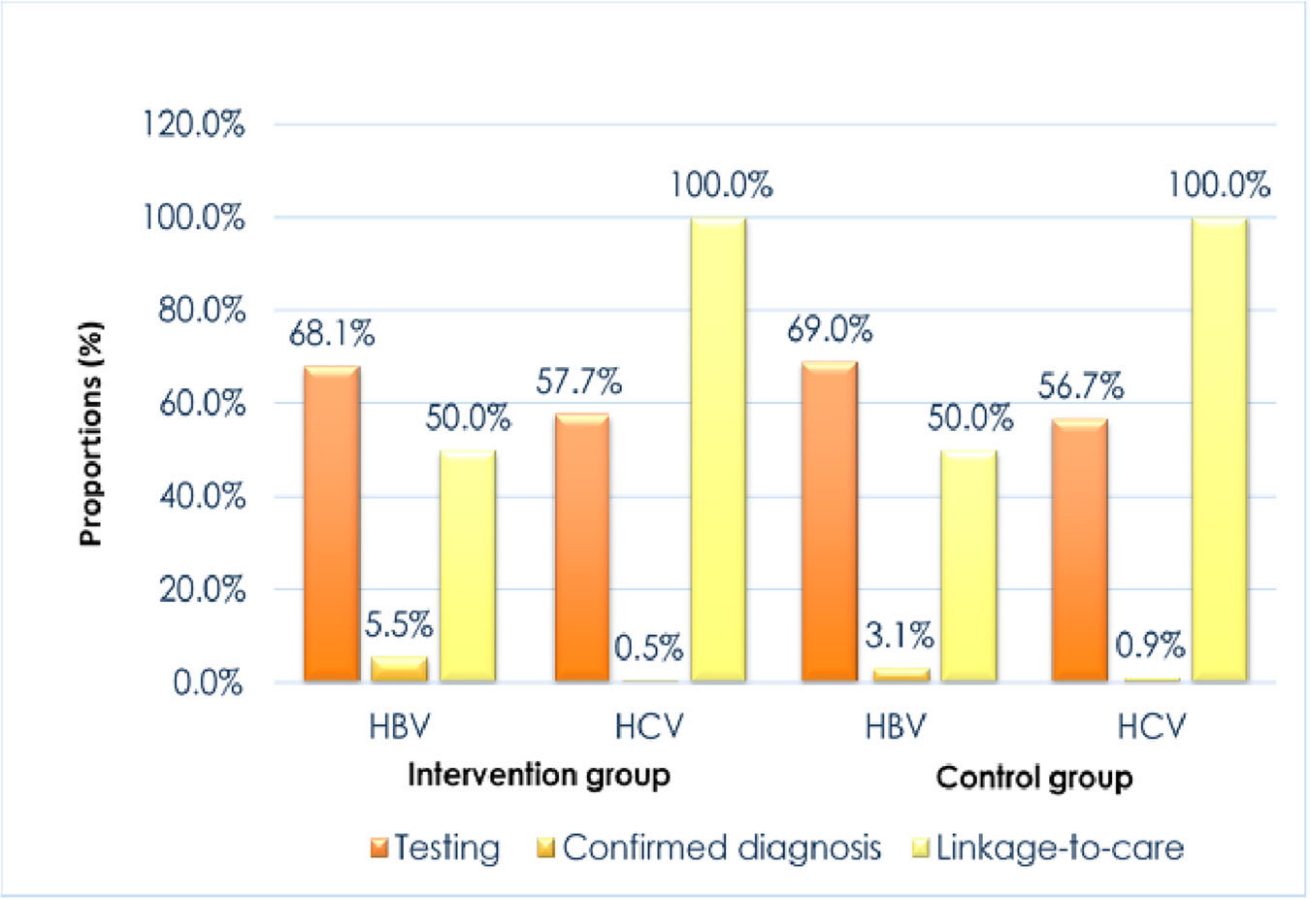
HBV and HCV testing uptake and linkage-to-care rates among primary care patients in urban China. Proportions of study participants in the intervention group vs. control group who accessed HBV and HCV testing and seeked treatment care post-testing at the family medicine and primary care clinic within the 4-weeks study period.

At the end of the 4-week study period, 14/256 (5.5%) of tested participants in the intervention group were HBV positive, and 1/217 (0.5%) was confirmed HCV positive. Among tested participants in the control group, 8/258 (3.1%) were HBV positive, and 2/212 (0.9%) were confirmed HCV positive. A total of 56% (14/25) of participants diagnosed with HBV and HCV were reachable by WeChat at follow-up. 50% (*n* = 11/22) of participants diagnosed with HBV and 100% (*n* = 3/3) of participants diagnosed with HCV self-reported receiving treatment (one at HKU-SZH hospital). Half (50%) of those who tested positive for HBV (*n* = 7/14) in the intervention group received treatment at HKU-SZH, two did not answer phone calls, one person reported not needing treatment per his physician’s report, and four people said that they did not even know they were HBV positive. Similarly, only 50% (*n* = 4/8) of the confirmed HBV positive participants in the control group self-reported receiving treatment at HKU-SZH. One person did not answer the phone call; 3 participants were unaware of their positive HBV test results (Fig. 2 below).

## Discussion

Our observed overall rate of confirmed HBV and HCV test uptake was higher than previously reported rates by a similar intervention to promote hepatitis testing among Chinese men who have sex with men (MSM)^28^. Although there was no significant difference in testing uptake rates between the intervention and control group, per-protocol analysis found that intervention group participants who saw all four intervention materials were 2–5 times more likely to test for HBV and HCV than those who did not see any. Half (50.0%) of HBV and all HCV diagnosed participants in this study reported successful linkage-to-care during a follow-up phone call, and hepatitis stigma reduced significantly among intervention group participants compared to the control group. Our study extends the literature by using crowdsourcing to enhance hepatitis testing in primary care settings, leveraging digital messages, and focusing on China. Our study addresses an urgent gap in expanding hepatitis testing by developing and evaluating an intervention to encourage HBV and HCV testing in primary care facilities. These findings may apply to up-scaling HBV and HCV programmes, especially in LMICs with high viral hepatitis burdens.

Our findings showed that hepatitis testing in primary care is feasible and acceptable to primary care users and could help expand hepatitis testing coverage in China. Among the 750 enrolled participants, 68.5% (514) tested for HBV, and 57.2% (429) tested for HCV. Our observed test uptake rates are higher than previously reported rates of 22.3% (124/556) HBV testing and 18.5% (103/556) HCV testing self-reported in a nationwide assessment among MSM in China^29^. Similar to our findings, a recent review of HBV and HCV testing strategies in the Europe^30^ and lessons learned from a previous model study conducted in the USA support the notion that hepatitis testing in primary care strategies is feasible with above-average acceptance various populations. As the COVID-19 outbreak has caused a lag in China’s achievement of 2020 targets towards the national agenda of curbing the transmission and reducing the hepatitis burden nationwide, promoting hepatitis testing services to get back on track is imminent.

However, a recent national survey of 200 primary care facilities in China found that only 15% had managed HBV in the preceding month, although 95% of primary care facilities were equipped to provide hepatitis testing services. Creating awareness about the availability of services remains urgent as a significant proportion of participants in our study cited not knowing where to get HCV testing as the main reason for not getting tested^9^. While active provider-initiated services could facilitate testing uptake, previous studies in China and Belgium found that most primary care providers do not offer hepatitis testing services due to limited knowledge and capacity, especially in conducting testing^31–33^. Fortunately, most primary care providers are eager for capacity-building as 84% of primary care providers in a cross-sectional study showed high interest in receiving training on HCV testing^34^. Hence, strategies to build the capacity of primary care providers to offer HBV and HCV testing services as part of routine are urgently needed to facilitate hepatitis testing service provision in primary care settings. Also, lessons from our study suggest that digital platforms like WeChat could effectively enable awareness creation and should be considered in hepatitis intervention designs.

In addition, digitally crowdsourced interventions could help increase HBV and HCV test uptake among primary care patients. Complete exposure to the four crowdsourced materials significantly increased the odds of HBV test uptake (aOR = 2.97, 95% CI: 1.02–8.26) and HCV test uptake (aOR = 5.10, 95% CI: 2.13–12.6) compared to not seeing any. Similar to this observation, using crowdsourcing was found to be non-inferior to health marketing strategies in improving condom use among MSM and transgender people by a previous study in China^35^. Furthermore, the capacity of crowdsourcing to improve STI testing promotion and engage communities in health campaigns has been demonstrated in multiple studies worldwide^26,36,37^. Hence, researchers should consider using crowdsourcing to tailor future hepatitis intervention programs to maximize their impact. It is also possible that the use of digital messages facilitated sharing and dissemination. The interactive nature of the digital intervention also enabled feedback from participants and facilitated community engagement. Thus, digital interventions could be an efficient strategy to create hepatitis services awareness and promote testing, especially in LMICS.

Our observation that exposure to the crowdsourced materials significantly reduced hepatitis stigma in the intervention group (HBV stigma adjusted beta = −0.15; HCV adjusted beta = −0.13) shows that crowdsourcing may decrease hepatitis stigma. Numerous studies have cited the fear of stigma as a leading cause of STI testing services, including hepatitis testing under-utilization among both high-risk populations like MSM and the general population^38,39^. Our study findings are similar to another RCT which found that crowdsourced messages decreased hepatitis B stigma (adjusted beta = −3.49, *p* = 0.01)^28^. Also, the results from a scoping review of 15 studies showed that crowdsourcing was a useful innovative tool for guiding the development of HIV and sexual health spurring interventions^38^. Therefore, utilizing crowdsourcing to develop tailored stigma reduction interventions across various population sub-types may represent an important strategy to be employed in subsequent research into elevating stigma barriers to viral hepatitis elimination.

There is, however, a need for innovative strategies to improve linkage-to-care. Although all confirmed HCV-positive participants (100%, 3/3), only 50% (7/14) of confirmed HBV-positive participants were successfully enrolled in treatment. Unfortunately, this is not a surprising finding as some studies have observed similar or lower linkage-to-care rates previously. Lower than our observed rate, only 9.5% (53) of participants reported visiting a physician after their hepatitis test in a similar RCT evaluating crowdsourcing strategies to improve hepatitis testing among MSM in China^28^. Alternatively, a study in Belgium found point-of-care testing for HBV to be associated with a higher linkage-to-care of 86% (6/7)^33^. The clinical protocol that requires treatment to be primarily undertaken by specialists and a low self-efficacy may hinder primary care providers from actively offering hepatitis care services. Therefore, a revision of protocols to fully integrate hepatitis care into primary care and the provision of adequate routine training for primary care providers may facilitate successful linkage-to-care in primary care.

Also, digital tools have shown great capacity in enabling patient engagement and enabling health services utilization for various diseases, including HIV and other STIs^16,40,41^. Hence the use of EHRs (like the “smart hospital” app of the HKU-SZH) could foster better patient management and referrals by ensuring prompt notification of confirmed positive patients to improve successful linkage-to-care. Moreover, the potential role for digital technology to assist RCT and clinical trials implementation in the future should be further researched.

Whiles China has strategies in place to expand hepatitis services delivery in primary care, findings from our study could inform the rollout and the upscale of implementation strategies for successful integration. Although our results showed that integration of HBV and HCV testing in primary care is feasible, introducing additional services in primary care may have extra consequences on facility management and staff workload. Factors like limited knowledge of hepatitis care among primary care providers, heavy workloads, a lack of chronic HBV-related guidelines, and limited human and financial resources in primary care settings could be potential barriers. Nonetheless, with generic antivirals now cheaply available and improved diagnostic techniques in primary care, routinely training healthcare service providers on hepatitis screening and management and providing tailored hepatitis care guidelines could significantly alleviate these barriers^42^. To address issues at the healthcare setting level, The Chinese Medical Association launched the first ‘Guideline for Primary Care of Chronic Hepatitis B (2020)’ in February 2021^43^. Additionally, the Hepatitis Foundation of New Zealand (“Foundation”) in a recent pilot found the use of a cloud-based national registry efficient in enabling general practitioners to monitor hepatitis patients in the community to reduce lost-to-follow-up rates (unpublished report). A similar tailored strategy could be adopted for implementation in China to enhance HBV and HCV services uptake in primary care settings.

Patient recruitment was negatively affected by the COVID-19 onset in China, which decreased our estimated total sample size of 1006 participants. To control local transmissions and prevent nationwide spread, the Chinese government in January 2020 expanded its Level One Public Health Emergency Response within mainland China. All provinces issued the highest level of emergency public health alerts and responses, with city-wide lockdowns that restricted movement and transportation. Many cities also underwent periodic lockdowns to contain sporadic localized outbreaks all year round. These control measures hindered access to facility-based services and drastically reduced facility visits in 2020. The fear of increased exposure at facilities due to the incidence of COVID-19 among health workers also reduced hospital visits and many people avoided seeking care during the study period fearing COVID-19 misdiagnosis and quarantine. Hence, fewer patients were available for recruitment during the study period, but the digital recruitment and study management enabled this study to be completed with a smaller sample size.

There are several potential reasons why we found no significant difference in HBV and HCV test uptake between the two groups. First, sharing of the intervention materials outside the intervention group may have contaminated our results^28^. However, we could not reassign participants based on self-reported exposure to adjust for the sharing effect in an as-exposed analysis because we had no systems in place to capture. Therefore, subsequent online trials that use information materials as interventions should be structured to restrict sharing or better capture and account for its impact. Additionally, recruiting participants from only one unit of the hospital may have restricted the generalizability of our study results. Second, follow-up time allotted for testing may have impacted outcome measures and linkage-to-care. As interventions may take longer to effect behavior change and linkage-to-care is an ongoing process, the four-week follow-up period in this study may have been too short to detect the effect of the intervention. Third, notifying participants of testing reimbursement prior to start may have promoted testing across both groups and overshadowed the effect of the intervention. Fourth, the self-reported proportions of exposure to intervention materials may be affected by social desirability bias and the possibility that seeing the materials does not automatically translate to actual reading of the full content. Regardless, our finding that exposure to all four materials significantly increased testing odds by two to five folds proves the effectiveness of the intervention, even when statistical power may be affected by a reduced sample size.

In conclusion, HBV and HCV testing in primary care are feasible and acceptable, and incorporating digitally crowdsourced interventions that utilize social media platforms could help improve hepatitis services uptake. Social media-based awareness creation strategies using digitally crowdsourced materials could effectively facilitate hepatitis test promotion and decrease hepatitis stigma, especially in resource-limited settings. However, linkage-to-care, especially among people newly diagnosed with HBV, remains far from the ideal coverage of 90%. Adopting eHRs may help improve linkage-to-care success for the two diseases, hence further studies are needed to evaluate the impact of tailored crowdsourcing interventions and eHRs in facilitating linkage-to-care in primary care settings.

## Methods

Before starting, the randomized controlled trial (RCT) was registered on the Chinese Clinical Trial Registry website (ChiCTR1900025771), and a detailed description of trial methods and study design was published^44^.

### Trial design

We carried out the study in the Department of Family Medicine and Primary Care (FMPC) unit of the University of Hong Kong-Shenzhen Hospital (HKU-SZH) in Shenzhen, China. The University of Hong Kong-Shenzhen Hospital has comprehensive clinical services, have over 1.52 million outpatient visits and 65,856 inpatient discharges annually, with hospital services easily accessible via digital mobile technology, and has a public WeChat group with >200,000 followers. The FMPC department reportedly attracts over 100,000 patient visits annually from Shenzhen in Guangdong province and nearby provinces in China.

Willing patients that visited the FMPC clinic who met the eligibility criteria were recruited to participate in the study by the onsite physicians. After providing written informed consent that outlined the study purpose and the participant’s rights and responsibilities in the Chinese language, participants provided an active unique mobile phone number and a WeChat account for enrollment. Enrolled participants received links to an online survey that collected baseline information including socio-demographic characteristics and HBV and HCV testing habits. Participants were required to add and follow the study’s official WeChat profile, which we used for all intervention materials (images and videos) distribution, study communication, and questionnaire dissemination.

HBV surface antigen (HBsAg) and HCV antibody (anti- HCV IgG) testing costs were refunded to participants via WeChat Pay (an online payment service) following confirmed testing by medical records at the FMPC at 4 weeks. After baseline survey and enrollment, participants were randomized to either receive: (1) crowdsourced intervention materials to promote HBV and HCV testing (intervention group); or, (2) standard-of-care services without hepatitis testing promotion materials (control group) for 4 weeks. Online follow-up surveys assessing primary and secondary outcomes of interest were sent to all participants via WeChat in the fourth week. Participants who did not test for HBV and HCV selected potential reasons for not getting tested from a predetermined list.

### Participants

Patients that were 30 years or older, resident in Shenzhen for the next one month, and not tested for HBV and HCV in the last 12 months were eligible. The age restriction was determined by evidence from previous studies conducted in China that found the risk of infection HBV and HCV infection and prevalence increased with age^45,46^. The study also observed an increase in reported incidence from passive HCV detection among individuals above 30 years^46^. In addition, findings from another China study showed that younger people (especially persons born from 1992 onwards) were more likely to be vaccinated and at lower risk of HBV infection^47^. Hence we hypothesized that the older population is more likely to benefit from interventions in hepatitis services expansion programs. Those who self-reported testing for HBV and HCV in the last 12 months, with known chronic HBV and HCV infection, undergoing treatment for HBV or HCV, undergoing complex medical treatment for other conditions, and pregnancy, were excluded.

### Intervention

The crowdsourcing process consisted of promotional materials development through a nationwide open contest in China. Representatives from 13 national and international organizations formed a steering committee and organized a nationwide open contest promoted on social media and the websites of partner organizations from February and May 2017. The open-contest solicited materials (like pictures, artworks, and one-minute videos) that sought to promote HBV and HCV testing and reduce hepatitis stigma. Of 168 eligible submissions collected, the two top-ranked images and two videos after the review and online poll process were selected as the intervention materials in this RCT (Supplementary Fig. 1). Details of the crowdsourcing process outlined elsewhere^28,48^, and copies of the final intervention materials are in Supplementary Fig. 2 (example of a crowdsourced video found in online version).

**Fig. 3:**
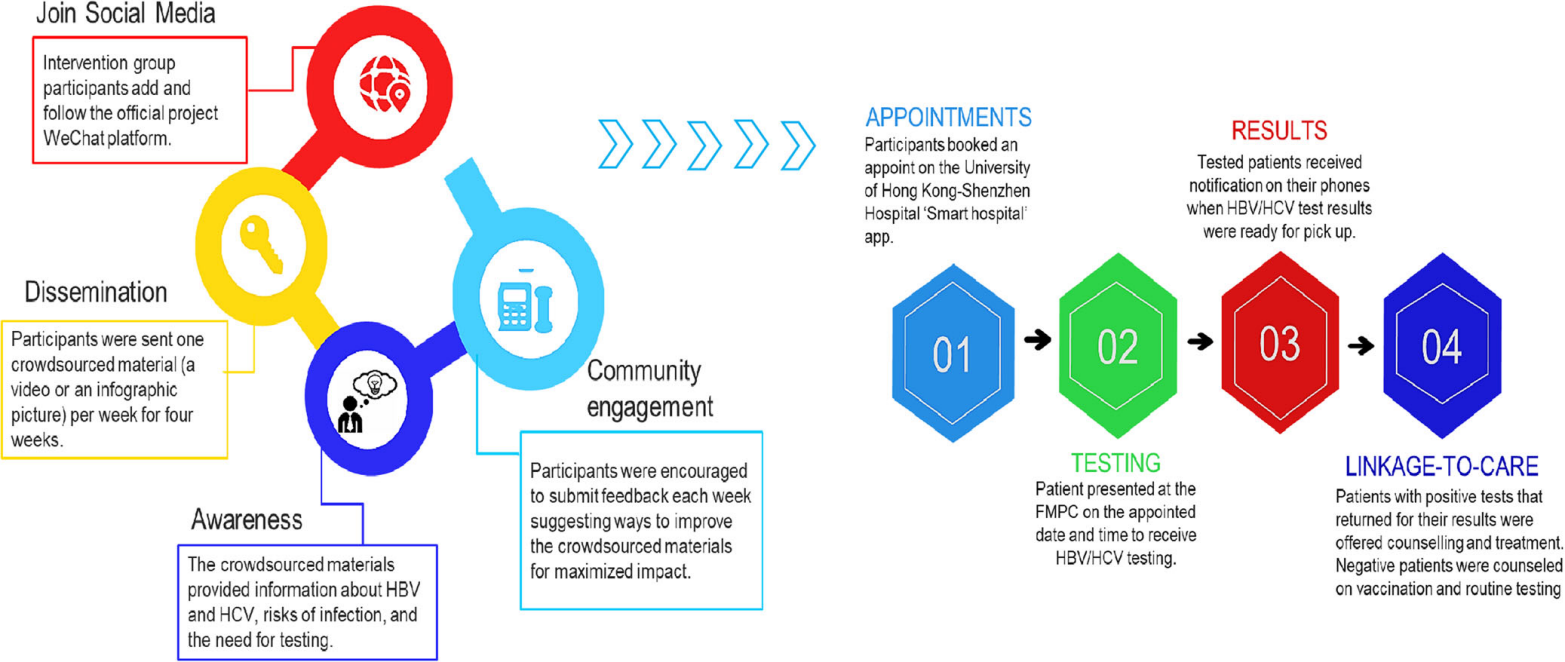
Digital dissemination of digitally crowdsourced intervention materials. Flowchart showing the process of distributing crowdsourced HBV and HCV testing promotion using social media and accessing hepatitis testing services using the ‘Smart hospital’ online application. Left diagram: digital dissemination cycle of the crowdsourced intervention materials; Right: Honk Kong University Shenzhen hospital online service application platform process.

The intervention consisted of two components: (1) a promotional component that involved the sharing of two videos and two images promoting HBV and HCV testing. Participants received one material each week for four weeks; (2) and an interactive session where participants submitted suggestions to better improve the intervention materials for better HBV and HCV testing promotion. Participants reflected on the intervention materials based on their expertise in healthcare, social media, or their personal experiences. All participants submitted at least a 50-character suggestions for improving the materials. The steering group members evaluated the submissions on: (1) potential to encourage HBV and HCV testing; (2) creativity; (3) potential to engage social media users; and (4) the feasibility of their use in clinical settings in Shenzhen. Top-ranked suggestions received up to 500 Chinese yuan (72.5 USD) as rewards. The control group participants received the standard healthcare services without any promotional materials and only received the baseline and follow-up surveys via WeChat. In the fourth week, all participants received reminders to test for HBV and HCV and complete the follow-up survey (see Fig. 3).

### Outcomes

The primary outcome was HBsAg and anti-HCV IgG test uptake within four weeks confirmed by medical records. Participant reports of testing were verified through the medical records of the FMPC clinic. Prevalence of hepatitis stigma among participants at baseline and at follow-up was measured using the Toronto Chinese hepatitis stigma scale with a Cronbach’s alpha of 0.9^39^ and confirmed linkage-to-care for participants with verified positive HBsAg and anti-HCV IgG tests were considered an independent component of secondary outcomes. Secondary outcomes also included the number of diagnosed participants receiving anti-HCV treatment versus those who declined treatment for any reason, and the number of participants diagnosed with chronic liver disease (including cirrhosis, liver failure, or hepatocellular carcinoma). See Supplementary Table 1 for detailed definitions of outcomes.

### Sample size

SAS software was employed in calculating the sample size. We assumed an estimated average anti-HCV seroprevalence of 3% among primary care users in China, a 35% testing rate based on conventional public health marketing methods (messages created by public health professionals), and that crowdsourcing improves testing rates by 10%. We estimated a sample size of 1006 patients (*n* = 503 in each arm) for the study to have a 90% power at an alpha risk of 5%^44^. We modified the CONSORT checklist to capture the COVID-19 effects on the overall study [CONSORT Checklist].

### Randomization and blinding

We randomly assigned participants to either the intervention or control group in a 1:1 ratio permuted blocks. The PROC PLAN and RANUNI functions in SAS software (Cary, North Carolina, USA) generated the randomization sequence. Allocation was sequential in the order of enrollment. Blinding was impossible as participants could easily predict their assignment based on whether they received the intervention materials, and the investigators were aware of the assignments during enrollment.

### Statistical methods

Baseline characteristics of all participants were summarized using descriptive statistics. Chi-square test, *T*-test, ANOVA were conducted where necessary to evaluate whether the crowdsourced materials intervention materials successfully increased HBV and HCV testing uptake compared to standard-of-care. Multivariable logistic regression results adjusted for potential confounders (age, sexual orientation, marital status, level of education, occupation, and income) was reported as odds ratios (OR) with 95% confidence intervals (95% CI).

Additionally, participants were sub-grouped based on whether they reported seeing all four intervention materials, one to three intervention materials, or no intervention materials during the 4-week study period in the per-protocol analysis. Missing data were considered in the intention-to-treat analysis using a ‘missing = failure’ approach. The approach included all participants lost to follow-up and assumed they did not achieve the primary and secondary outcomes. No variable had >15% missing data and we performed multiple imputations using R (MICE package). Linear regression was used to examine the effect of the intervention on hepatitis stigma between the intervention and control group. SPSS v23 and R-studio v3.6.2 were used for all statistical analyses and *p* < 0.05 was statistically significant.

### Reporting summary

Further information on research design is available in the Nature Research Reporting Summary linked to this article.

## Supplementary information

Supplementary Materials

Reporting Summary

Consent Form (in Simplified Chinese)

Study protocol (in Simplified Chinese)

An example of a crowdsourced video used in the present study

## Data Availability

The data generated during this study that support the reported findings are available from the corresponding author upon reasonable request.
