# Supplementary material for: A randomized controlled trial enhancing viral hepatitis testing in primary care via digital crowdsourced intervention
Source: NPJ Digit Med. 2022 Jul 19;5:95. doi: 10.1038/s41746-022-00645-2 (PMC9296450; doi:10.1038/s41746-022-00645-2)
Supplement: Supplementary file 1 — Supplementary Materials [file 41746_2022_645_MOESM1_ESM.pdf]

SUPPLEMENTARY FILES LEGEND

Supplementary Table 1: Detailed definitions of all primary and secondary outcomes.

Supplementary Table 2: Summary showing the reasons why 642 primary care patients did not test for HBV and HCV within the four weeks study period, China, 2019-2021.

Supplementary Table 3: Per-protocol analysis evaluating the impact of a crowdsourced intervention on hepatitis testing uptake among 270 primary care users in urban China, 2019-2021.

Supplementary Table 4: Results if ANOVA test comparing the impact of crowdsourcing interventions on hepatitis stigma reduction within the intervention group by level of exposure

Supplementary Figure 1: Development of images and videos to promote HBV and HCV testing through a public crowdsourcing challenge contest.

Supplementary Figure 2: Sample snips showing images from the Crowdsourced HBV and HCV testing promotion materials.

Supplementary Table 1. Primary and Secondary outcomes with definitions

**Primary Outcomes**	**Definition**
Confirmed HBV testing uptake	Participants that complete HBV testing as confirmed by medical records at HKU-SZH or other public facility in Shenzhen within 4 weeks of enrollment
Confirmed HCV testing uptake	Participants that complete HCV testing as confirmed by medical records at HKU-SZH or other public facility in Shenzhen within 4 weeks of enrollment
**Secondary Outcomes**	
Self-reported HBV testing uptake	Participants that complete HBV testing within 4 weeks of enrollment, self-reported in follow-up survey
Self-reported HCV testing uptake	Participants that complete HCV testing within 4 weeks of enrollment, self-reported in follow-up survey
Followed-up for HCV confirmatory testing	Participants that follow-up with a provider at HKU-SZH for HCV RNA diagnostic testing, as confirmed by medical records at HKU-SZH or other public facility in Shenzhen
Self-reported follow-up for HCV confirmatory testing	Participants that follow-up with a provider at HKU-SZH or other care facilities in China for HCV RNA confirmatory testing, self-reported in follow-up survey
Linkage-to-care	Participants that have a confirmed HBV and HCV diagnosis that self-reported initiating anti-HBV and anti-HCV treatment at HKU-SZH Department of Gastroenterology, confirmed by medical records at HKU-SZH or other public facility in Shenzhen
Hepatitis stigma	Self-reported notions and believes about persons living with hepatitis and how they should be treated as rated on the hepatitis stigma scale.

Supplementary Table 2. Summary showing the reasons why 642 primary care users enrolled in an online randomized controlled trial to evaluate a crowdsourced intervention did not test for HBV and HCV in China, 2019-2021.

	**Intervention（N=310）**		**Control (N=332）**		**χ^2**	**P-value**
	n	%	n	%		
**HBV**						
HBV testing site					2.564	0.464
The UKU-Shenzhen Hospital	247	79.7	253	76.2		
Other hospitals in Shenzhen	5	1.6	3	0.9		
Shenzhen CDC	2	0.6	0	0		
Other health services in Shenzhen	2	0.6	2	0.6		
Reasons for not testing (multiple choice)						
Cannot afford the medical bill	3	1.0	4	1.2	0.118	0.732
No Shenzhen medical insurance	5	1.6	14	4.2	4.241	0.039
HBV is not curable	6	1.9	1	0.3	0.064	0.056
Don't consider themselves at risk	33	10.6	37	11.1	0.159	0.690
Do not know where to do the test	7	2.3	11	3.3	0.819	0.365
No time to do the test	38	12.3	37	11.1	0.075	0.784
Worried might be discriminated against	3	1.0	4	2.3	1.000	0.516
Don't think HBV is a serious problem.	12	3.9	10	3.0	0.262	0.609
Other	36	11.6	42	12.7	0.393	0.531
Sought medical care after receiving HBV results	125	40.3	135	40.7	0.629	0.428
Discussed HBV testing with a doctor in the past four weeks	185	59.7	195	58.7	0.059	0.808
Vaccinated against HBV in the past four weeks	25	8.1	20	6.0	1.024	0.312

**HCV**						
HCV testing site					2.001	0.736
The UHK-Shenzhen Hospital	212	68.4	208	62.7		
Other hospitals in Shenzhen	2	0.6	2	0.6		
Community health centers in Shenzhen	0	0.0	1	0.3		
Other health services in Shenzhen	1	0.3	1	0.3		
Medical institution outside of Shenzhen	1	0.3	0	0.0		
Sought medical care after receiving HCV results	97	31.3	110	33.1	2.087	0.149
Reasons for not testing						
Cannot afford the medical bill	2	0.6	5	1.5	0.451	0.254
No Shenzhen medical insurance	6	1.9	5	1.5	3.441	0.064
HBV is not curable	3	1.0	0	0.0	0.112	0.112
Don't consider themselves at risk	51	16.5	53	16.0	0.033	0.855
Don’t know where to do the test	21	6.8	21	6.3	0.056	0.812
No time to do the test	43	13.9	44	13.3	0.060	0.807
Worried might be discriminated against	3	1.0	1	0.3	0.357	0.286
Don't think HCV is a serious problem.	17	5.5	32	9.6	4.114	0.043
Other	40	12.9	45	13.6	0.063	0.802
Discussed HCV testing with a doctor in the past four weeks	137	44.2	144	43.4	0.044	0.834

Note: p-value < 0.05 statistically significant.

Supplementary Table 3. Per-protocol analysis evaluating the impact of a crowdsourced intervention on hepatitis testing uptake among 270 primary care users in urban China, 2019-2021.

	HBV testing		HCV testing	
	**OR (95% CI0**	**aOR (95% CI)**	**OR (95% CI)**	**aOR (95% CI)**
Multiple Imputation analysis (N=268)				
**Marital Status**				
Single	ref	ref	ref	ref
Married/Lived with a partner	0.38 (0.11-1.01)	0.29 (0.08-0.87)*	0.72 (0.34-1.44)	0.68 (0.29- 1.51)
Divorced/Separated	0.23 (0.04-1.36)	0.22 (0.03-1.67)	0.84 (0.20-4.30)	0.96 (0.21-5.39)
Widowed	0.17 (0.01-4.21)	0.03 (0.00-1.13)*	0.16 (0.01-1.79)	0.07 (0.00-1.14)
**Intervention Exposure**				
Saw none of the materials	ref	ref	ref	ref
Saw some of the materials [a]	0.89 (0.30-2.59)	0.87 (0.25-3.00)	1.80 (0.69-4.81)	2.03 (0.69-6.17)
Saw all the materials	2.33 (0.90-5.61)	2.87 (0.99-7.97)	4.38 (1.99-9.85)***	5.22 (2.17-12.97)***
Multiple Imputation analysis (N=270)				
**Marital Status**				
Single	ref	ref	ref	ref
Married/Lived with a partner	0.36 (0.10-0.96)	0.26 (0.07-0.76)*	0.77 (0.37-1.51)	0.66 (0.29-1.43)
Divorced/Separated	0.23 (0.04-1.33)	0.20 (0.03-1.51)	0.91 (0.22-4.63)	0.95 (0.21-5.29)
Widowed	0.17 (0.01-4.12)	0.02 (0.00-0.94)*	0.17 (0.01-1.93)	0.06 (0.00-0.99)
**Intervention Exposure**				

Saw none of the materials	ref	ref	ref	ref
Saw some of the materials [a]	0.89 (0.30-2.59)	0.86 (0.24-2.95)	1.80 (0.69-4.81)	1.88 (0.64-5.66)
Saw all the materials	2.26 (0.87-5.42)	2.84 (0.97-7.87)	4.20 (1.91-9.42)***	4.87 (2.04-11.99)***

Notes: [a] Had seen some of the materials meaning that participants looked at promotional materials from one to three weeks; *p value < 0.05; *** p value < 0.001. Adjusted variables include age, sex, sexual orientation, marital status, education, occupation, and monthly income.

Supplementary Table 4. ANOVA test comparing the impact of crowdsourcing interventions on hepatitis stigma reduction within the intervention group by level of exposure (N=270).

**Variables**	**Had not seen any of materials (n=30)**	**Had seen some of materials (n=38)**	**Had seen all materials (n=202)**	**p-value**
**Stigma of HBV**				
Average score	2.29	2.53	2.33	0.247
**Stigma of HCV**				
Average score	2.36	2.44	2.36	0.839

Crowdsourcing Flowchart

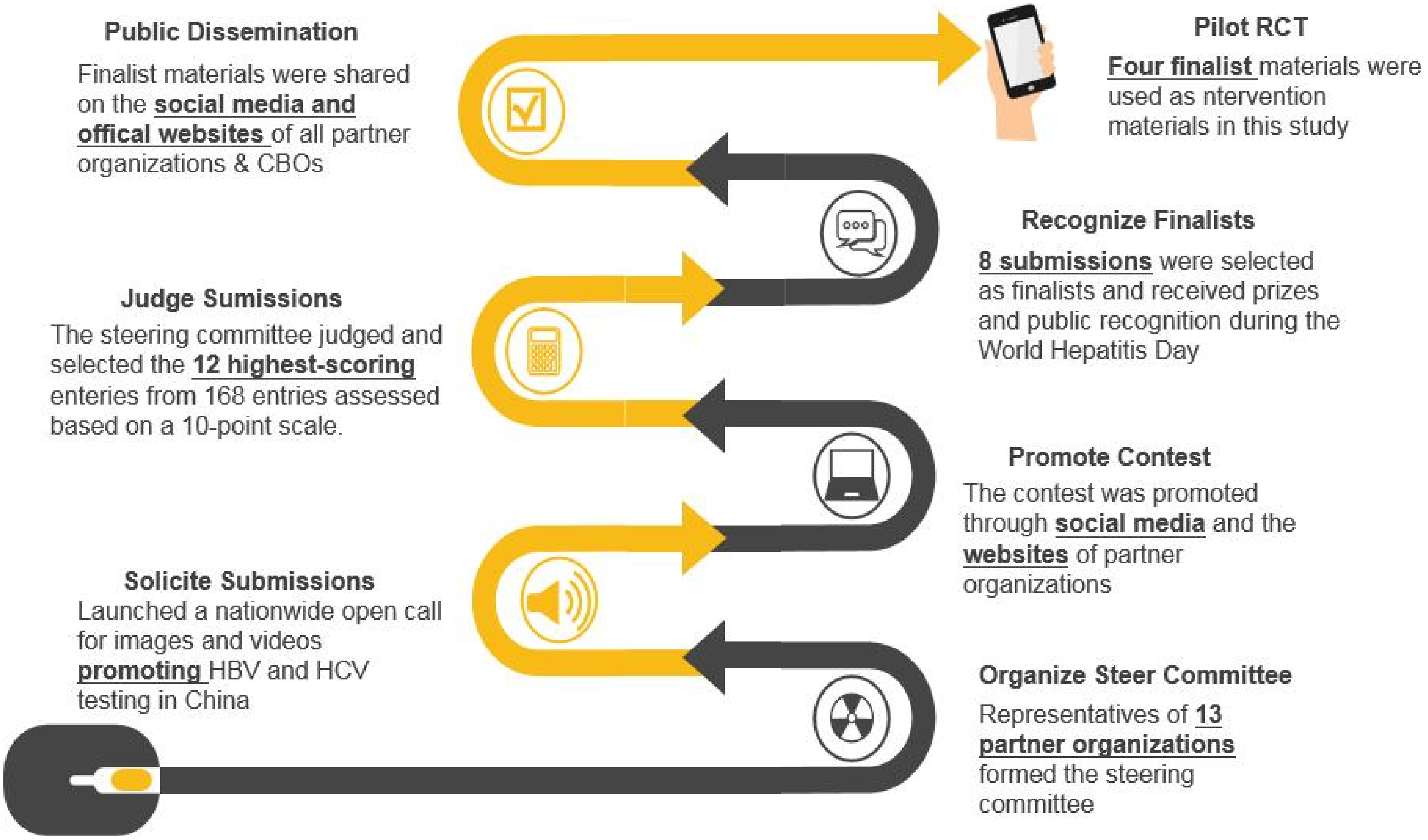

Supplementary Figure 1 Development of images and videos to promote HBV and HCV testing through a nationwide crowdsourcing open call. The process followed these six steps: (1) organizing a steering committee, (2) soliciting entries, (3) promoting the contest, (4) judging entries, (5) recognizing excellent entries, and (6) sharing entries.

HBV and HCV Testing Promotion Images

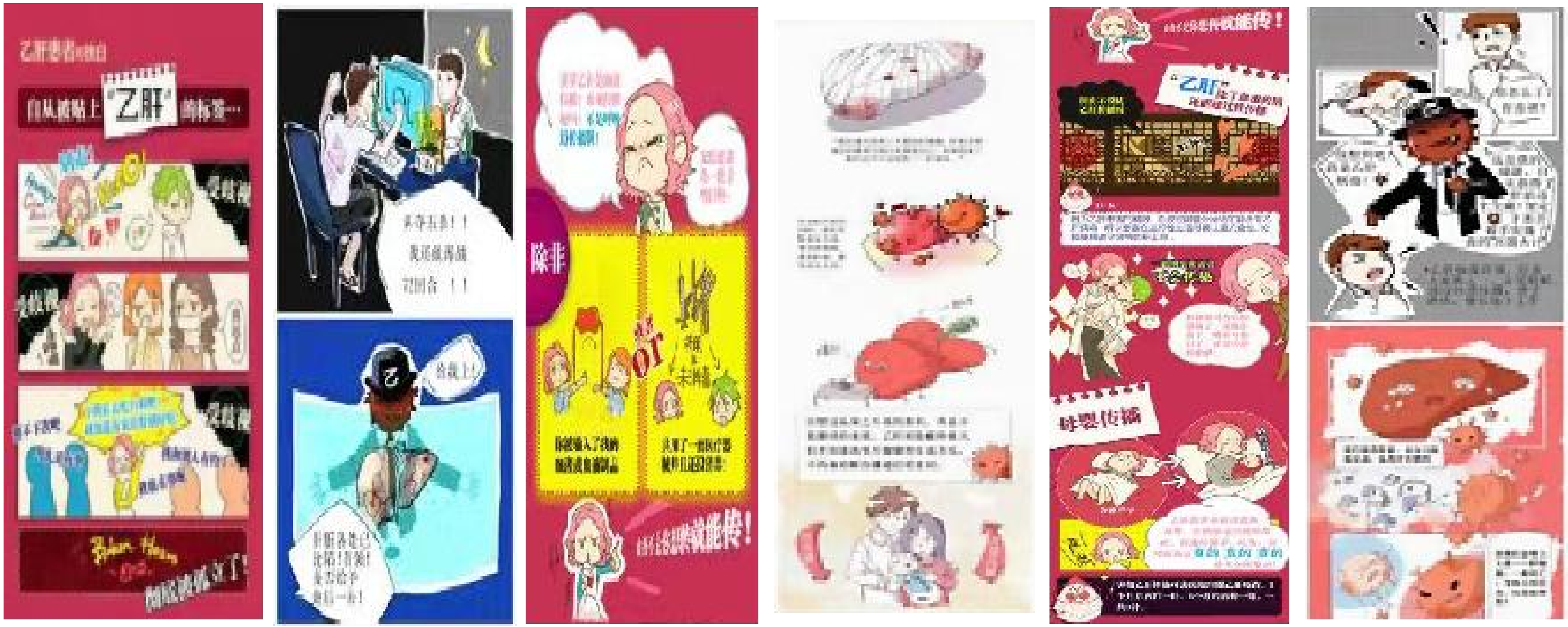

Supplementary Figure 2 Sample snips showing images from the Crowdsourced HBV and HCV testing promotion images used as intervention materials in this study.
