# Supplementary material for: A randomized controlled trial enhancing viral hepatitis testing in primary care via digital crowdsourced intervention
Source: NPJ Digit Med. 2022 Jul 19;5:95. doi: 10.1038/s41746-022-00645-2 (PMC9296450; doi:10.1038/s41746-022-00645-2)
Supplement: Supplementary file 2 — Reporting Summary [file 41746_2022_645_MOESM2_ESM.pdf]

nature portfolio

Corresponding author(s): Dr. Wai-Kay Seto

Last updated by author(s): May 30, 2022

Reporting Summary

Nature Portfolio wishes to improve the reproducibility of the work that we publish. This form provides structure for consistency and transparency in reporting. For further information on Nature Portfolio policies, see our Editorial Policies and the Editorial Policy Checklist.

Statistics

For all statistical analyses, confirm that the following items are present in the figure legend, table legend, main text, or Methods section.

n/a	Confirmed	
☐	☒	The exact sample size (n) for each experimental group/condition, given as a discrete number and unit of measurement
☐	☒	A statement on whether measurements were taken from distinct samples or whether the same sample was measured repeatedly
☐	☒	The statistical test(s) used AND whether they are one- or two-sided<br>*Only common tests should be described solely by name; describe more complex techniques in the Methods section.*
☐	☒	A description of all covariates tested
☐	☒	A description of any assumptions or corrections, such as tests of normality and adjustment for multiple comparisons
☐	☒	A full description of the statistical parameters including central tendency (e.g. means) or other basic estimates (e.g. regression coefficient) AND variation (e.g. standard deviation) or associated estimates of uncertainty (e.g. confidence intervals)
☐	☒	For null hypothesis testing, the test statistic (e.g. F, t, r) with confidence intervals, effect sizes, degrees of freedom and P value noted<br>*Give P values as exact values whenever suitable.*
☒	☐	For Bayesian analysis, information on the choice of priors and Markov chain Monte Carlo settings
☒	☐	For hierarchical and complex designs, identification of the appropriate level for tests and full reporting of outcomes
☒	☐	Estimates of effect sizes (e.g. Cohen's d, Pearson's r), indicating how they were calculated

Our web collection on statistics for biologists contains articles on many of the points above.

Software and code

Policy information about availability of computer code

Data collection	Microsoft Excel
Data analysis	SPSS v23 and R-studio v3.6.2

For manuscripts utilizing custom algorithms or software that are central to the research but not yet described in published literature, software must be made available to editors and reviewers. We strongly encourage code deposition in a community repository (e.g. GitHub). See the Nature Portfolio guidelines for submitting code & software for further information.

Data

Policy information about availability of data

All manuscripts must include a data availability statement. This statement should provide the following information, where applicable:

- Accession codes, unique identifiers, or web links for publicly available datasets
- A description of any restrictions on data availability
- For clinical datasets or third party data, please ensure that the statement adheres to our policy

The data generated during this study that support the reported findings are available from the corresponding author upon reasonable request.

Field-specific reporting

Please select the one below that is the best fit for your research. If you are not sure, read the appropriate sections before making your selection.

☐ Life sciences ☒ Behavioural & social sciences ☐ Ecological, evolutionary & environmental sciences

For a reference copy of the document with all sections, see nature.com/documents/nr-reporting-summary-flat.pdf

Behavioural & social sciences study design

All studies must disclose on these points even when the disclosure is negative.

Study description	We conducted a randomized controlled study which collected quantitative data on socio-graphic characteristics, hepatitis B and C virus testing uptake, and assessed hepatitis stigma among primary care patients using pre-tested questionnaires.
Research sample	We carried out the study in the Department of Family Medicine and Primary Care (FMPC) unit of the University of Hong Kong-Shenzhen Hospital (HKU-SZH) in Shenzhen, China. The University of Hong Kong-Shenzhen Hospital has comprehensive clinical services, have over 1.52 million outpatient visits and 65,856 inpatient discharges annually, with hospital services easily accessible via digital mobile technology, and has a public WeChat group with >200,000 followers. The FMPC department reportedly attracts over 100,000 patient visits annually from Shenzhen in Guangdong province and nearby provinces in China.<br><br>Patients that were 30 years or older, resident in Shenzhen for the next one month, and not tested for HBV and HCV in the last 12 months were eligible. The age restriction was determined by evidence from previous studies conducted in China that found the risk of infection HBV and HCV infection and prevalence increased with age.
Sampling strategy	We employed a convenience sampling method and the sample size was estimated using SAS software. We assumed an estimated average anti-HCV seroprevalence of 3% among primary care users in China, a 35% testing rate based on conventional public health marketing methods (messages created by public health professionals), and that crowdsourcing improves testing rates by 10%. We estimated a sample size of 1006 patients (n = 503 in each arm) for the study to have a 90% power at an alpha risk of 5%.<br><br>Unfortunately, the recruited sample size of 750 was smaller due to the impact of COVID-19 preventive measures in China during the study period (November 2019 - June 2021). These effects have been duly discussed in our article and reported as part of the CONSORT statement
Data collection	Data was collected using pre-tested baseline and follow-up surveys hosted online, the WeChat app, hospital electronic health record system, pen, book, laptop and follow-up phone calls. The surveys were self-administered and the researchers were aware of the participant allocations.
Timing	November 2019 to June 2021
Data exclusions	No data was excluded in our intention-to-treat analysis.
Non-participation	20 people declined participation, 12 people were exempted for not completing the baseline survey and 3 participants were excluded for not providing informed consent. 114 participants were lost-to-follow up at the end of the study.
Randomization	We randomly assigned participants to either the intervention or control group in a 1:1 ratio permuted blocks. The PROC PLAN and RANUNI functions in SAS software (Cary, North Carolina, USA) generated the randomization sequence. Allocation was sequential (1:1 ratio) in the order of enrollment. Blinding was impossible as participants could easily predict their assignment based on whether they received the intervention materials, and the investigators were aware of the assignments during enrollment.

Reporting for specific materials, systems and methods

We require information from authors about some types of materials, experimental systems and methods used in many studies. Here, indicate whether each material, system or method listed is relevant to your study. If you are not sure if a list item applies to your research, read the appropriate section before selecting a response.

Materials & experimental systems

n/a	Involved in the study	
☒	☐	Antibodies
☒	☐	Eukaryotic cell lines
☒	☐	Palaeontology and archaeology
☒	☐	Animals and other organisms
☐	☒	Human research participants
☐	☒	Clinical data
☒	☐	Dual use research of concern

Methods

n/a	Involved in the study	
☒	☐	ChIP-seq
☒	☐	Flow cytometry
☒	☐	MRI-based neuroimaging

Human research participants

Policy information about studies involving human research participants

Population characteristics	See Above
Recruitment	See Above
Ethics oversight	The institutional review board of the Hong Kong University Shenzhen Hospital (HKU-SZH) approved the study (Ref no: hkuszh201888). All participants provided written informed consent that highlighted their right to opt-out of the study at any point prior to participation in the study.

Note that full information on the approval of the study protocol must also be provided in the manuscript.

Clinical data

Policy information about clinical studies

All manuscripts should comply with the ICMJE guidelines for publication of clinical research and a completed CONSORT checklist must be included with all submissions.

Clinical trial registration	The study was registered in the Chinese Clinical Trial Registry (ChiCTR1900025771) on September 9, 2019. Available from: http://www.chictr.org.cn/showproj.aspx?proj=42788
Study protocol	The published study protocol is available from: https://pubmed.ncbi.nlm.nih.gov/32615951/
Data collection	See Above
Outcomes	The primary outcome was HBsAg and anti-HCV IgG testing uptake within four weeks confirmed by medical records. Participant reports of testing were verified through the medical records of the FMPC clinic.<br>Prevalence of hepatitis stigma among participants at baseline and at follow-up was measured using the Toronto Chinese hepatitis stigma scale with a Cronbach's alpha of 0.9 39 and confirmed linkage-to-care for participants with verified positive HBsAg and anti-HCV IgG tests were considered an independent component of secondary outcomes. Secondary outcomes also included the number of diagnosed participants receiving anti-HCV treatment versus those who declined treatment for any reason, and the number of participants diagnosed with chronic liver disease (including cirrhosis, liver failure, or hepatocellular carcinoma).
