# Supplementary material for: A randomized controlled trial enhancing viral hepatitis testing in primary care via digital crowdsourced intervention
Source: NPJ Digit Med. 2022 Jul 19;5:95. doi: 10.1038/s41746-022-00645-2 (PMC9296450; doi:10.1038/s41746-022-00645-2)
Supplement: Supplementary file 3 — Consent Form (in Simplified Chinese) [file 41746_2022_645_MOESM3_ESM.docx]

**《众包法在中国基层促进病毒性肝炎及相关治疗：随机对照研究》**

**知情同意书，伦理委员会号码：hkuszh201888**

我们真诚邀请您参加此项研究。此项目已通过香港大学深圳医院伦理委员会的批准。本着自愿参与的原则，您可随时退出，也可以拒绝回答问卷的任何问题。下文为关于该项研究的细节介绍:

**我们为什么开展此项研究？** 大多数中国人都不知道自己是否被感染乙肝/丙肝，因为感染后多年都可能没有任何症状，在不知情下，又把乙肝/丙肝病毒传给他们的亲友，或者发现感染时已经发展到无法治愈的程度，如肝硬化，肝功能衰竭或肝癌等。这个研究最重要的意义是要让更多的人在无症状时接受检测，在病情恶化之前接受治疗，保护自己和他人免受感染。

**我们通过此研究想了解什么？** 此项研究的目的是了解“众包”是否可以帮助医生增加深圳居民的乙肝/丙肝检测。众包是我们公开地让大众解决问题，然后与社区分享解决方案的过程。本研究中，我们将使用在众包比赛中创建的材料来鼓励受试者接受乙肝/丙肝检测。您如果符合以下所有条件，您即可参加本研究：

1. 您年龄超过30岁; 2. 居住在深圳市一个月以上; 3. 您是中国公民。

如果有以下情況，您不能参加本研究：

1. 您已经怀孕或计划在本研究期间怀孕; 2. 您在过去12个月接受过乙肝/丙肝检测；3.您已知患有慢性乙肝或丙肝。

**参加此项研究，您需要做些什么？** 在加入研究后，我们会让您添加为微信好友。您将要通过手机完成一个基线问卷，与我们进行沟通。您将在注册后收到第一份问卷，并在四周后收到最后一份问卷。您可能会被问及某些敏感问题，您不必回答任何让您感到不安的问题。此问卷大约需要10-15分钟完成。一半参与者会随机分配观看众包创作的旨在推广肝炎检测的视频和图片，即干预组。另一半不将收到任何材料。您自己不能选择参与哪一组。

如果您在干预组，我们会在2-3周内通过微信把资料直接发送到您的手机上。您通过微信观看图片和视频之后，将被要求提供有关如何改进这些资料的建议。每次观看材料以及提出建议所花费的时间不会超过5分钟。我们每周与您联系不会超过两次。我们还会征求您的同意，以匿名方式与其它参与者分享您的反馈。提供获奖建议的参与者将获得现金奖励。

如果您在参与研究的四周内，在深圳的任何诊所／医院接受了乙肝/丙肝检测，我们将为您报销检测费用。您必须将检测结果的照片发送给我们，以确认您进行了检测。在完成基线调查三到四周后，您将通过微信收到后续调查问卷。随访调查将询问您是否接受了乙肝/丙肝检测，以及您对肝炎的态度。我们还会询问您是否与家人或朋友分享了我们提供的材料等。

研究阶段结束之后，研究人员可能会邀请您向我们提供您参与研究的经历和反馈。这一步可能通过电话，当面访谈，或小组访谈完成。您可以接受或拒绝。您的决定不会影响您参与研究的资格或港大医院对您的待遇。

**该项研究共招募多少人？** 如果您愿意参与此项研究并符合参与标准，您将会是本研究的1006名参与者之一。本研究将从2019年进行到2021年。

**参与该项研究可能带来哪些好处？**如果您接受丙肝/乙肝检测，我们将为您报销所有检测费用：包括乙肝检测费用135元，丙肝检测费用为28元，最高总计163元人民币。完成研究的所有程序之后，您还将获得额外的100元人民币作为您对我们研究做出贡献的报酬。接受乙肝和丙肝检测的参与者也可以了解自己的感染情况，并及时获得治疗。

**参与该研究的可能风险和不适有哪些？** 我们将要求您提供敏感信息，当您分享此类个人信息时，您可能会感到尴尬，担心，或紧张，我们将对您的个人资料严格保密。如果您在研究过程中接受肝炎检测，我们将询问您的检测结果。因此您可能会暴露与您的乙肝和丙肝检测以及感染状况的相关信息，但只限于研究团队。您可能会担心研究人员排斥您或者与其他人讨论您的检测结果，也可能担心您的名声或者地位由于您的肝炎状态被发现而受到损害。我们会采取相应的安全措施，保障所有参与者的个人隐私（详情见下述），使任何个人、雇主或者保险机构获得这些信息的可能性维持在极小程度。于新发感染案例，也不会有任何疾病干预措施。患者的家人或伴侣也不会被联系。

**您能从该项研究中收获什么?**如果您需要乙肝的治疗，药物，或肝炎专科随访，国家保险將涵盖这些费用。但丙肝的治疗费用必須自己承担。您可以选择在其他医院或诊所测试乙、丙肝。

**您需要付费吗？** 如果您决定在香港大学深圳医院接受检测，您需要自己挂号，安排自己往返医院的交通费用。我们会提供部分检测的费用。由于您必须花时间进行测试，您可能会错过工作或产生其他费用。我们无法承担这些费用。如在其他医院接受治疗，本项目並不提供任何經濟上的支援或醫療疗责任。

**我们如何保护您的隐私？**研究人员将通过微信访问有关参与者的个人和其他信息，微信将仅用于传递信息。我们将使用您的香港大学深圳医院就诊卡号验证您的肝炎医疗案卷。我们会小心保管您的姓名或私人信息，以保护您的隐私。您提供的任何个人信息只有研究或医疗人员才能取用。

参与者完成调查后，所有数据都会直接输入电脑；数据机密性将通过数据的安全加密、传播和储存的保护。数据需要在服务器的防火墙内通过密码才能获取。只有主要研究者和特定资深项目成员可以获得存储个人信息渠道的密码。参与者电话的IP地址在任何时候都不会被收集。上网产生的痕迹不会被用来跟踪参与者的活动。

审核员，检查员/监督员，机构审查委员会/独立伦理审查委员会和其他监管机构将被允许审查任何参与者的原始医疗记录，以验证此研究的程序，和／或与本研究相关的法律和法规的程序。 我们承诺采取一切适当措施，以尽量减少违反保密规定的风险。我们不发表或公布任何可识别个人身份的信息。只有在法律要求的情况下才能放弃保密保护(即涉嫌虐待儿童，怀疑虐待老人，威胁对自己或他人造成直接伤害)。根据国际上的法律，临床试验的描述可见于www.clinicaltrials.gov。该网站不会记录您的信息，仅仅会记录全部统计数量。您可以随时登录该网站查阅有关本研究的信息。

**作为参与者，我的责任是什么？** 如果您有任何不明白的事情，或者您在任何时候对研究有疑虑，您有责任提问。您有责任报告参与时遇到的任何副作用。您有责任及时准确地完成我们研究中的所有步骤。您还有责任准确，诚实地报告您的医疗信息。

**如果关于本次研究，您还有其他问题：**您有权利询问关于本项研究的任何问题。如果您有任何问题、抱怨或疑虑，您可以联系本研究的主要研究者。如果您有任何意料之外的反应，未遵循本试验的指示或没有达到我们的参与标准，研究人员有权力停止您的参与。如果研究团队了解到可能影响您参与决定或参与资格的信息，我们将立即通知您或您的法律代表。

**如果您对研究参与者的权利还不了解？** 伦理委员会会审查所有涉及人类志愿者的研究，以便保护参与者的权利与利益。如果您对该项研究还有其他问题或顾虑，可通过电话（+86 18033050703）或微信（+86 18033050703）联系本研究的工作人员（周一至周六上午8点至下午5点30分）。

如果您理解并同意参加此项网上调查，请勾选下方的“同意”。我们非常感谢您的参与！

- 同意
- 不同意
- 我愿意在研究结束时提供我的参与经历和反馈。

签名：________________________________

日期：________________________________
