# Supplementary material for: A randomized controlled trial enhancing viral hepatitis testing in primary care via digital crowdsourced intervention
Source: NPJ Digit Med. 2022 Jul 19;5:95. doi: 10.1038/s41746-022-00645-2 (PMC9296450; doi:10.1038/s41746-022-00645-2)
Supplement: Supplementary file 4 — Study protocol (in Simplified Chinese) [file 41746_2022_645_MOESM4_ESM.pdf]

众包法在中国基层促进病毒性肝炎的测试及相关治疗：随机对照研究临床研究方案

研 究 单 位：香港大学深圳医院全科医学科

主要研究者：黄志威

一、研究题目：

众包法在中国基层促进病毒性肝炎的测试及相关治疗：随机对照研究

二、研究背景：

乙肝病毒(HBV)和丙肝病毒(HCV)正日益成为全球卫生问题，估计分别有近3 亿和7110 万人感染。如不及时治疗，HBV 和HCV 慢性感染都可导致重型肝病。HCV 预计将成为肝硬化、肝衰和肝细胞癌的主要病因，到2025 年，HCV 相关死亡率将增加3 倍。在中国感染乙肝病毒的8600 万人中，仅约19%的人了解这些。同样，中国有3000 万人的丙肝抗体呈阳性，仅占感染人群的一小部分。未接受治疗的感染者可能在感染后的20 至30 年内出现严重并发症。乙肝疫苗接种，早期检测和直接使用抗病毒治疗可有效控制乙肝，可治愈丙肝。尽管检测很重要，但仅一小部分HBV 和HCV 患者知道自己的状况、接受随访及抗病毒治疗。促进检测及相关治疗是有效遏制病毒性肝炎在中国传播的关键。然而，在复杂的医疗体系中，患者不知道如何找到合适的筛查及相关治疗。此外，对HBV 患者的病耻感和歧视，以及公众对该病了解不足，可能是患者寻求HBV 治疗的障碍。因此，自愿性的乙肝丙肝检测率很低，使中国初级保健诊所的检验设施没有充分利用。最近的一个对肝炎检测和治疗意愿的全国性的调查中，发现一半的社康中心有丙肝检验设备，且多数医护人员也认识到这项服务的益处，但即使病人主动寻求检测，也仅三分之一的医护人员这样做。医护人员在肝炎诊断治疗方面没有得到充分培训，忽略了这类病人。明确初级

保健在提供肝炎保健方面的作用的政策和指导方针，可以改善中国普通人群对病毒性肝炎的检测及治疗的认识。众包是开发有效干预措施以应对病毒性肝炎公共卫生挑战的一种很有潜力的方法，同时提高公众对HBV 和HCV 的认识。众包是让非专家和专家解决问题，然后与社区分享解决方案的过程。通过众包开发的材料已被证明可以提高艾滋病毒(HIV)的检测率，且与循证材料一样有效。

三、研究目的：

病毒性肝炎尽管检测很重要，但只有一小部分HBV 和HCV 患者知道自己的肝炎状况，并接受随访及抗病毒治疗。促进检测和随后的相关治疗是有效遏制病毒性肝炎在中国传播的关键。促进检测和随后与治疗服务的联系对于有效遏制乙肝病毒和丙肝病毒在中国的传播至关重要。众包是开发有效干预措施以应对病毒性肝炎公共卫生挑战的一种极有潜力的方法，同时也能提高公众对HBV 和HCV 的认识。我们相信，众包将帮助我们在中国开发出一种方法来增加乙肝病毒和丙肝病毒筛查，从而使更多的乙肝和丙肝病毒感染者得到必要的治疗，并降低中国的乙肝和丙肝相关发病率和死亡率。

具体目标1:比较众包干预与标准医疗对中国基层医疗患者HBV 和HCV 检测的影响

具体目标 2:比较众包干预与标准医疗对中国 HBV 和 HCV 相关治疗的影响。

四、研究设计：

参与者中国HCV 抗体阳性率估计在2.2%到3.7%之间[Lavanchy Clin Microbiol infection 2011, Wei L, Lok AS, Gastroenterology 2014]。由于它不如HBV 流行,我们将基于我们对HCV 的计算来进行。因此，如果使用3%的人口流行率估计值，招募的人口中可能有9%具有抗丙肝病毒阳性反应。标准组按市场营销方法（marketing methods）的测试率约为35%。如果我们保守地假设众包将测试率提高了10%，加上90%的功耗和5%的甲级风险（alpha risk），那么我们将需要750 名患者的样本量(每组375 人，n=375)，估计有402 名参与者同意进行丙肝病毒测试。根据估计的9%的阳性率,我们提出的研究将确定36 例抗丙肝病毒阳性患者。27 名患者(75%)预计HCV RNA 阳性，需要治疗。招生工作将持续24 个月，目标是招收750名参与者。参加者将会亲自及在网上招募。主要的现场招聘地点将是FMPC 候诊室，以及HKU-SZH 的内窥镜室，我院内窥镜室每年要做22000 次内窥镜检查。在美国，在内窥镜室进行丙肝病毒筛查已被证明可增加三倍。有关研究的资料亦会透过香港大学深圳医院公共关系科公布。参加者可选择直接透过网上报名或随后亲自现场报名参加。纳入标准为30 岁以上，因为这是美国的“婴儿潮”一代(即1945-1965 年出生)，是慢性丙肝感染的高危人群。尽管中国的高危人群可能有所不同，但有证据表明，报告的丙肝病毒发病率随着年龄的增长而增加。

在中国,30岁以上的人由于被动的丙肝病毒检测而报告的发病率有所上升参加者如自行报告，或其在HKU-SZH 的记录中曾测试HBV

或HCV 抗体阳性，则将被排除在外;在研究之前已知他们患有慢性乙肝或丙肝病毒感染;或者他们有严重的并发症比如恶性肿瘤;或者他们是第一个进入决赛的人或者是我们众包竞赛的第一个团队进入干预的均被排除在外。

五、研究流程:

第一阶段:众包竞赛与线上干预的开发

举办一个公开的众包竞赛，为乙肝和丙肝病毒测试及相关治疗创作极具冲击力的概念和内容。这项比赛将透过社交媒体推广至本地社区中心、社康组织、病人权益组织、医院义工及以及高校学生等， 如深圳大学等大专院校的学生或全科的护士学生。入围名单将由专家评审小组从提交的材料中选出，专家评审小组成员包括丙肝患者、公共卫生官员、医生和营销专家。在我们的研究的第二阶段中，最后的决赛者或决赛者团队将把他们的获胜理念整合到一个单一的在线干预方案中，作为干预。

第二阶段:随机对照试验(RCT)

为了测试参与者的招募数量、参与者粘性、调查有效性及预防干扰干预组使用方法的可行性，将在全面RCT 之前首先进行试点研究。该试验将使用以前的众包材料来进行肝炎检测。我们预计，媒体宣传活动的公开特性会增加对照组参与者因公开分享而受到干预的风险。我们的试点项目将测试防止干扰的方法的可行性和有效性。

该试验将在RCT 启动前试行三个月。

将招募 50 名参与者参与试点研究，在线或亲自参与。招募完成后，参与者将完成基线调查并随机分组。干预组将通过互联网的一个网上链接接触众包干预材料，而对照组的参与者将通过一个网上链接接触一般的标准保健资料。标准保健资料是港大深圳医院现有的关于 HBV 和 HCV 的宣传材料，例如全科的 HCV 宣传手册。陪同参与者的来访者将被排除在我们的研究之外。在后续的调查中，我们会要求干预组的参与者提供关于众包材料的反馈，这些反馈将会被用来持续改进我们的干预材料。试验将评估这个重复过程的可行性，以改进我们的众包干预，并将检查我们的调查的有效性和文化适宜性。

试点研究完成后，本次研究将招募共计750名参与者，基于参与者中国HCV 抗体阳性率估计在2.2%到3.7%之间。[Lavanchy Clin Microbiol infection 2011, Wei L, Lok AS, Gastroenterology 2014]。由于它不如HBV 流行，我们将基于我们对HCV 的计算来进行。因此，如果使用3%的人口流行率估计值，招募的人口中可能有9%具有抗丙肝病毒阳性反应。标准组按市场营销方法的测试率约35%。如果我们保守地假设众包将测试率提高了10%，加上90%的功耗和5%的甲级风险（alpha risk)，那么我们需要750 名患者的样本量。一个双组的 RCT 将评估用众包的、由同行设计的在线干预的有效性，同时与标准的HBV 和HCV测试的接受度相对比。

参与者将在同意入组（签署知情同意书）后第一周及第四周完成相关问卷（该问卷仅涉及询问是否进行乙肝/丙肝检测；以及参与者

对肝炎的态度。我们还会询问参与者是否与家人或朋友分享了我们提供的材料等）。为鼓励参与者尽可能进行检测，参与者在研究的四周内，在深圳的任何诊所 / 医院接受了乙肝/丙肝检测，我们将为参与者报销检测费用。

据推测，在促进中国普通大众接受HBV和HCV检测方面，众包在线干预将优于标准保健和不暴露于社交媒体的方式。

第三阶段:在香港大学深圳医院的乙肝/丙肝相关治疗

根据自愿咨询测试(VCT) 服务模式，鼓励招募的参与者参加港大深圳医院的匿名保密乙肝/丙肝检测和咨询服务。在测试前和测试后，参与者均要填写相关问卷。基于乙肝将以促进行为改变为最终目标， VCT 服务还将解决该特定个体HBV/HCV感染的潜在原因，并鼓励预防行为和定期体检的习惯。我们会邀请有兴趣的参加者参加业内培训计划，培训他们成为同行业内教育工作者，并让他们具备在业内进行乙型肝炎/丙型肝炎教育的知识和技能。

经 HCV RNA检测确认感染的患者可转至香港大学深圳医院消化内科肝病门诊进行进一步诊断检查，并进行直接抗病毒治疗的讨论。我们将讨论基于sovaldi 的治疗方法，如果可行的话，它将被基于epclusbased的治疗方法所取代。患者随后将接受持续病毒学反应监测。根除成功后，肝硬化或进展期纤维化患者将继续在港大深圳医院肝病门诊随访，非进展期纤维化患者将转诊全科进行随访。

六、受试者在研究中的利益：

完成基线调查的参与者及完成后续调查的参与者将获得额外100元补偿。在测试期间，HBV 筛查试验(HBsAg)和HCV 筛查试验(血清抗HCV 抗体)以及确诊试验(血清HCV RNA，用于对HCV 呈阳性的个人)将在HKU-SZH 免费为干预和对照组受试者提供。

七：受试者在研究中的风险：

因为围绕这个话题的病耻感很普遍，如果参与者自己公开我们关于HBV（和HCV)的干预材料，他们可能会经历社会性的病耻感。其他风险很小。

八、项目主要实现的技术指标及期望的结果

假设1:在中国基层医疗人群中，在促进HBV 和HCV 检测使用方面，众包干预将优于标准医疗干预。

假设2:在中国疾病医疗人群中，在促进HBV 和HCV 的相关治疗方面，众包干预将优于标准医疗。

主要结果是:注册4 周后,RCT 两组参与者完成的HBsAg 和抗hcv 筛查试验的数量。

次要结果是：入组后4 周自我报告HBV 或HCV 检测但未与HKU-SZH 记录确认的人数；参加HKUSZH自愿咨询测试（VCT）服务的人数;随访HBV 或HCV 测试验证了的参与者人数;参加HKU-SZH 培训计划的人数;诊断为HBV 或HCV 的参与者继续接受抗HBV 或抗HCV 治疗的人数，与因各种原因无法负担或拒绝接受治疗的人数相比;完全

治愈丙肝病毒感染或实现持续乙肝病毒抑制的参与者人数，与完成治疗前退出的人数相比;确诊为慢性肝病的参与者人数;参与慢性肝病随访的患者人数。

九、数据处理方法及数据的保存

初步分析：使用描述性分析总结参与者的基线特征。初步分析将评估在中国普通大众和目标人群中增加HBV/HCV 检测使用率方面，基于互联网的众包干预方案是否优于标准保健方案。使用95%二项比例置信区间(binomial proportion confidence interval, CI)分别计算干预组和对照组中确认的HBV/HCV 检测的参与者的比例。干预组和对照组在检测吸收比例上的差异将用相应的Wald 95% CI 来计算。还将进行z 检验，以比较干预组和对照组接受测试的比例。如果概率差异的95% CI 完全大于零，那么干预将被宣布优于对照。如果测试比例低于预期，以致计算Wald CI 的统计假设不满足，那么就会计算出概率差异的确切95% CI。干预对HBV/HCV 检测率的影响将首先使用包括随访失败的参与者在内的分析方法进行评估，而随访失败的参与者被认为没有进行HBV/HCV 检测。还将进行一项暴露分析（as-exposed analysis)，根据参与者自己报告的干预暴露程度来分配他们。

效果测量修正分析：效果测量修正分析将评估干预对HBV/HCV 检测率的影响是否因以下基线因素而变化：既往医疗服务使用行为、对医生和卫生保健系统的信任度、对基层保健服务的认识和参与度、是否为HCV 高危人群。

缺失数据计划：任何在随访的任何阶段不再参与的人将被认定在研究期间没有达到主要和次要的结果。如果少于15%的参与者没有得到结果，分析人员将使用完全个案的方法来处理。如果结果是中途退出者≥15%，分析将使用多重替代法（multiple imputation)。

次要分析：还将比较对照组和干预组的参与者在达到次要结果的比例方面的差异：需要转诊进行HCV 确诊性测试的人数、接受丙肝病毒确诊的人数、参加香港大学深圳医院 HBV/HCV 治疗的随访人数、获得丙肝病毒治愈的人数、达到乙肝病毒持续性抑制的人数。在安全的在线调查平台Wenjuanxing 上收集基线和随访调查数据。HBV/HCV 检测结果在HKUSZH的随访，研究团队将使用姓名和人口统计信息追踪患者信息。所有研究数据将在传输前进行加密，并存储在安全的服务器中，并且只能通过研究团队知道的登录信息访问。

十、研究中主要到的问题：

1.因为围绕这个话题的病耻感在社会上很普遍，如果参与者自己公开我们关于HBV（和HCV)的干预材料，他们可能会经历社会上的病耻感。其他道德风险很小。

2.我们不认为本研究中的干预措施会使参与者面临任何重大的道德风险，所有参与者均是在自愿的基础上获得免费的测试和免费的后续治疗。在治疗的选择上，我们的三甲医院的专科医生会和病人共同讨论，最终在医疗法律的框架下决定药物的选择。

3.调查中所有涉及的个人信息，均只有研究团队知晓，并做到严

格保密。这些信息，除了研究以外，不做任何其他用途。我们还将寻求医院审查委员会的批准。

4.我们的所有调查，是在安全的在线调查平台Wenjuanxing 上收集基线和随访调查数据的。对于HBV/HCV 检测结果在HKU-SZH 的随访，研究团队将使用姓名和人口统计信息追踪患者信息。所有研究数据将在传输前进行加密，并存储在安全的服务器中，并且只能通过研究团队知道的登录信息访问。所有的过程都是在患者的知情同意下进行的。

5.我们的研究团队均承诺该申请书内容真实与准确；承诺该研究符合相关伦理原则；承诺该研究符合医院的相关政策，并遵循医院伦理委员会标准操作规程要求，接受伦理的持续监督与审查；承诺研究结束以后将保存研究资料至少3 年以上。

主要研究者：黄志威

2021年12月01日

基线调查问卷

1. 您的年龄是？ [填空题] *

____

2. 您的性别是？ [单选题] *

○男性

○女性

○其他 (例如跨性别者)

3. 您的婚姻状况是？ [单选题] *

○未婚

○已婚/同居

○离婚/分居

○丧偶

4. 您的性取向是? [单选题] *

○异性恋

○同性恋/双性恋

○不确定/其他

5. 您的最高受教育程度是？ [单选题] *

○初中或以下

○完成高中或技校/专科学校

○本科毕业

○硕士、博士或以上

6. 您的主要职业是？ [单选题] *

○学生/家庭主妇

o自雇

o受雇

o公务员

o待业

o退休

7. 您的月收入(税后)是多少？ [单选题] *

o5,000 元以下

o5,001 – 10,000 元

o10,001 – 15,000 元

o15,001 – 20,000 元

o20,000 元以上

以下问题有关医疗服务的使用经验

8. 您最常看病的地点是？ [单选题] *

o香港大学深圳医院

o深圳其他医院

o深圳社区卫生服务中心

o深圳疾控中心

o深圳其他的卫生服务

o深圳市以外地区

9. 您在港大深圳医院看病用什么付费的？ [单选题] *

o国家医疗保险

o公司团体医疗保险

o其他/个人商业医疗保险

o自费

10. 您目前有固定的全科医生吗？ [单选题] *

o有

o没有

11. 对医务人员的信任度[矩阵单选题] *

	完全不同意	不同意	中立	同意	非常同意
我相信医务人员给我提供了最好的治疗	o	o	o	o	o
我相信医务人员在尽最大的努力为我着想	o	o	o	o	o
我相信如果在治疗过程中有任何失误，医务人员会如实告诉我	o	o	o	o	o

以下问题将询问您与乙肝的经验以及态度

12. 您以前听说过乙肝吗？ [单选题] *

o有

o没有

13. 您进行过乙肝检测吗？ [单选题] *

o有

o没有

14. 您上一次检测乙肝是什么时候？ [单选题] *

o从来没检测过乙肝/不知道

o过去 1 到 5 年内

o5 年多以前

15. 有否接种过乙肝疫苗？ [单选题] *

○从未/不知道是否接种过乙肝疫苗

○有，过去 1 到 5 年内

○有，5 年多以前

16. 请回答您是否同意以下关于乙肝的观点。以下问题没有对错[矩阵单选题] *

	非常不同意	不同意	中立	同意	非常同意
为了保护大众，乙肝感染者应被隔离	○	○	○	○	○
我和乙肝感染者一起吃饭会觉得不舒服	○	○	○	○	○
如果乙肝感染者从事与儿童密切接触的工作，他们可能会给儿童带来感染风险	○	○	○	○	○
乙肝感染者一定做过不好的事情，被感染是活该的	○	○	○	○	○
我不想雇佣乙肝感染者	○	○	○	○	○
我不想和乙肝感染者成为朋友	○	○	○	○	○
如果我感染上了乙肝，我会觉得我让我的亲人失望了	○	○	○	○	○
如果我感染上了乙肝，没人会想跟我交往	○	○	○	○	○

以下问题将询问您与丙肝 的经验以及态度。

17. 您以前听说过丙肝吗？ [单选题] *

o有

o没有

18. 您进行过丙肝检测吗？ [单选题] *

o有

o没有

19. 您上一次检测丙肝是什么时候？ [单选题] *

o从来没检测过丙肝/不知道

o过去 1 到 5 年内

o5 年多以前

20. 您是否同意以下关于丙肝的观点，以下问题没有对错。[矩阵单选题] *

	非常不同意	不同意	中立	同意	非常同意
为了保护大众，丙肝感染者应被隔离	o	o	o	o	o
我不想雇佣丙肝感染者	o	o	o	o	o
如果我感染上了丙肝，会觉得需要把这感染的信息隐藏起来	o	o	o	o	o
如果我感染上了丙肝，没人会想跟我交往	o	o	o	o	o

21. 当您发生性关系时，会使用安全套吗？ [单选题] *

o每次都用

o大部分时候都用

o很少用

o从来不用

22. 您是否有检测过艾滋病？ （自检或在诊所/医院检测） [单选题] *

o从来没有检测过

o有,我是艾滋病阳性

o有，我是艾滋病阴性

o有，没有取得检测结果/我忘记了结果

23. 请回答您是否有过以下的行为。[矩阵单选题] *

	是	否
我曾经以性来交换金钱,礼物或其他好处	o	o
我曾经注射过兴奋类药品	o	o
我有身体穿孔(如打耳洞等）.纹身,或曾经接受过针刺疗法。	o	o
在 1991 年前，我献过血或注射过血液制品（包括血浆）	o	o
在 1991 年前，我做过手术	o	o
我正在或曾经接受过血液透析的治疗	o	o
我曾经接受过性传播感染疾病的治疗 （如淋病/白浊,衣原体感染，梅毒）	o	o
我家族中有乙肝病史	o	o
我家族中有丙肝病史	o	o

联系信息

24. 您的手机号码是多少？（添加手机号码的目的仅为调查所用。整个过程中，我们都会严格保障参与者的隐私。） [填空题] *

____

25. 您的微信账号是什么？(添加微信账号的目的仅为调查所用。整个过程中,我们都会严格保障参与者的隐私。) [填空题] *

____

感谢您的参与！

我们真诚邀请您到香港大学深圳医院接受免费的乙肝与丙肝检测。

您也可以选择在其他医院或诊所进行检测。

随访调查问卷 C

1.您的手机号码是多少？（请保持与基线调查时的号码一致）

（添加手机号码的目的仅为匹配基线调查所用。整个过程中，我们都会严格保障参与者的隐私。） [填空题] *

____

2.您的微信账号（非微信昵称）是多少？

（添加微信的目的仅为匹配基线调查所用。） [填空题] *

____

下面的问题询问您有关乙肝检测的经历。

3.在过去四星期内，您检测乙肝了吗? [单选题] *

○有（转到第 4-6 题） (请跳至第 4 题)

○没有（转到第 7 题） (请跳至第 7 题)

4.您的乙肝检测结果是什么？ [单选题] *

○阳性

○阴性

○不确定

○没有收到结果

5.在拿到乙肝检测结果后，您去看过医生吗？ [单选题] *

○看过

○没有

6.您是在哪里检测乙肝？ [单选题] *

○香港大学深圳医院

○深圳其他医院

o深圳社区卫生服务

o深圳疾控中心

o其他深圳的卫生服务

o深圳市外的卫生服务

7.您为什么没有做乙肝检测？（可以多选） [多选题] *

□付不起医疗检测费用

□没有深圳的医保

□认为乙肝无法治愈

□不认为自己有感染的风险

□不知道去哪做检测

□没时间检测

□担心被检测出来乙肝会被歧视

□不觉得乙肝是个严重的问题

□其他 _________________

8.在过去四星期内，您跟医生讨论过关于乙肝检测吗？ [单选题] *

o有过

o没有

9.在过去四星期内，您有接种乙肝疫苗吗？ [单选题] *

o有过

o没有

10.您的乙肝状况属于以下哪种？ [单选题] *

o没有检测过乙肝

o目前未感染乙肝

o以前感染过乙肝，但已治愈

o以前打过疫苗

○没有打过疫苗，也没有被感染过

○不清楚

11.以下问题将询问您与乙肝的态度。请回答您是否同意以下观点。以下问题没有对错。[矩阵单选题] *

	非常不同意	不同意	中立	同意	非常同意
为了保护大众，乙肝感染者应被隔离	○	○	○	○	○
我和乙肝感染者一起吃饭会觉得不舒服	○	○	○	○	○
如果乙肝感染者从事与儿童密切接触的工作，他们可能会给儿童带来感染风险	○	○	○	○	○
乙肝感染者一定做过不好的事情，被感染是活该的	○	○	○	○	○
我不想雇佣乙肝感染者	○	○	○	○	○
我不想和乙肝感染者成为朋友	○	○	○	○	○
如果我感染上了乙肝，我会觉得我让我的亲人失望了	○	○	○	○	○
如果我感染上了乙肝，没人会想跟我交往	○	○	○	○	○

下面的问题询问您有关丙肝检测的经历。

12.在过去四星期内，您检测丙肝了吗? [单选题] *

○有（转到 13-15 题） (请跳至第 13 题)

○没有（转到 16 题） (请跳至第 16 题)

13.您的丙肝检测结果是什么? [单选题] *

○阳性

○阴性

○不确定

○没有拿到结果

14.在拿到丙肝检测结果后，您去看过医生吗？ [单选题] *

○有过

○没有

15.您是在哪里检测丙肝？ [单选题] *

○香港大学深圳医院

○深圳其他医院

○深圳社区卫生服务

○深圳疾控中心

○其他深圳的卫生服务

○深圳市外的卫生服务

16.您为什么没有做丙肝检测？（可以多选） [多选题] *

□付不起医疗检测费用

□没有深圳的医保

□认为丙肝无法治愈

□不认为自己有感染的风险

□不知道去哪做检测

□没时间检测

□担心被检测出来丙肝会被歧视

□不觉得丙肝是个严重的问题

□其他 ________________

17.请问在过去四星期内，您曾经跟医生讨论过关于丙肝检测的问题吗？ [单选题] *

○有过

○没有

18.以下问题将询问您与丙肝的态度。请回答您是否同意以下观点。[矩阵单选题] *

	非常不同意	不同意	中立	同意	非常同意
为了保护大众，丙肝感染者应被隔离	○	○	○	○	○
我不想雇佣丙肝感染者	○	○	○	○	○
如果我感染上了丙肝，会觉得需要把这感染的信息隐藏起来	○	○	○	○	○
如果我感染上了丙肝，没人会想跟我交往	○	○	○	○	○

19.在过去的四周内，您有收到过肝炎相关的视频或者图片吗？ [单选题] *

○有

○没有

随访调查问卷 I

1.您的手机号码是多少？（请保持与基线调查时的号码一致）

（添加手机号码的目的仅为匹配基线调查所用。整个过程中，我们都会严格保障参与者的隐私。） [填空题] *

____

2.您的微信账号（非微信昵称）是多少？

（添加微信的目的仅为匹配基线调查所用。） [填空题] *

____

下面的问题询问您有关乙肝检测的经历。

3.在过去四星期内，您检测乙肝了吗? [单选题] *

○有（转到第 4-6 题） (请跳至第 4 题)

○没有（转到第 7 题） (请跳至第 7 题)

4.您的乙肝检测结果是什么？ [单选题] *

○阳性

○阴性

○不确定

○没有收到结果

5.在拿到乙肝检测结果后，您去看过医生吗？ [单选题] *

○看过

○没有

6.您是在哪里检测乙肝？ [单选题] *

○香港大学深圳医院

○深圳其他医院

o深圳社区卫生服务

o深圳疾控中心

o其他深圳的卫生服务

o深圳市外的卫生服务

7.您为什么没有做乙肝检测？（可以多选） [多选题] *

□付不起医疗检测费用

□没有深圳的医保

□认为乙肝无法治愈

□不认为自己有感染的风险

□不知道去哪做检测

□没时间检测

□担心被检测出来乙肝会被歧视

□不觉得乙肝是个严重的问题

□其他 ________________

8.在过去四星期内，您跟医生讨论过关于乙肝检测吗？ [单选题] *

o有过

o没有

9.在过去四星期内，您有接种乙肝疫苗吗？ [单选题] *

o有过

o没有

10.您的乙肝状况属于以下哪种？ [单选题] *

o没有检测过乙肝

o目前未感染乙肝

o以前感染过乙肝，但已治愈

o以前打过疫苗

○没有打过疫苗，也没有被感染过

○不清楚

11.以下问题将询问您与乙肝的态度。请回答您是否同意以下观点。以下问题没有对错。[矩阵单选题] *

	非常不同意	不同意	中立	同意	非常同意
为了保护大众，乙肝感染者应被隔离	○	○	○	○	○
我和乙肝感染者一起吃饭会觉得不舒服	○	○	○	○	○
如果乙肝感染者从事与儿童密切接触的工作，他们可能会给儿童带来感染风险	○	○	○	○	○
乙肝感染者一定做过不好的事情，被感染是活该的	○	○	○	○	○
我不想雇佣乙肝感染者	○	○	○	○	○
我不想和乙肝感染者成为朋友	○	○	○	○	○
如果我感染上了乙肝，我会觉得我让我的亲人失望了	○	○	○	○	○
如果我感染上了乙肝，没人会想跟我交往	○	○	○	○	○

下面的问题询问您有关丙肝检测的经历。

12.在过去四星期内，您检测丙肝了吗？ [单选题] *

○有（转到第 13-15 题） (请跳至第 13 题)

○没有（转到第 16 题） (请跳至第 16 题)

13.您的丙肝检测结果是什么? [单选题] *

o阳性

o阴性

o不确定

o没有拿到结果

14.您是在哪里检测丙肝？ [单选题] *

o香港大学深圳医院

o深圳其他医院

o深圳社区卫生服务

o深圳疾控中心

o其他深圳的卫生服务

o深圳市外的卫生服务

15.在拿到丙肝检测结果后，您去看过医生吗？ [单选题] *

o有过

o没有

16.您为什么没有做丙肝检测？（可以多选） [多选题] *

□付不起医疗检测费用

□没有深圳的医保

□认为丙肝无法治愈

□不认为自己有感染的风险

□不知道去哪做检测

□没时间检测

□担心被检测出来丙肝会被歧视

□不觉得丙肝是个严重的问题

□其他 ________________

17.请问在过去四星期内，您曾经跟医生讨论过关于丙肝检测的问题吗？ [单选题] *

○有过

○没有

18.以下问题将询问您与丙肝的态度。请回答您是否同意以下观点。[矩阵单选题] *

	非常不同意	不同意	中立	同意	非常同意
为了保护大众，丙肝感染者应被隔离	○	○	○	○	○
我不想雇佣丙肝感染者	○	○	○	○	○
如果我感染上了丙肝，会觉得需要把这感染的信息隐藏起来	○	○	○	○	○
如果我感染上了丙肝，没人会想跟我交往	○	○	○	○	○

19.以下的问题将询问您过去 4 周内是否看过发送给您的四个肝炎视频或者图片。[矩阵单选题] *

	看过	没看过
您看过第一个标题为“远离肝炎，从检测做起”的视频吗？	○	○
您看过第二个标题为“对抗乙肝&丙肝升级打怪史”的漫画图片吗？	○	○
您看过第三个标题为“乙肝患者的独白”，了解肝炎的漫画吗？	○	○

您看过第四个标题为“拒绝肝扰，一肝二净”的视频吗？	○	○

20.看过视频/图片后, 您是否做了以下？(可以多选) [多选题] *

□跟家族人/朋友/其他人分享了视频/图片

□自己在网络上搜索了乙肝/丙肝的知识

□跟他人谈了关于乙肝/丙肝检测的事

□以上都没有
